# Genetic dissection of Sharka disease tolerance in peach (*P. persica* L. Batsch)

**DOI:** 10.1186/s12870-017-1117-0

**Published:** 2017-11-03

**Authors:** Marco Cirilli, Laura Rossini, Filippo Geuna, Francesco Palmisano, Angelantonio Minafra, Tiziana Castrignanò, Stefano Gattolin, Angelo Ciacciulli, Anna Rosa Babini, Alessandro Liverani, Daniele Bassi

**Affiliations:** 1 Department of Agricultural and Environmental Sciences (DISAA), University of Milan, via Celoria 2, Milan, Italy; 2 Parco Tecnologico Padano, via Einstein, Loc. C.na Codazza, Lodi, Italy; 3 Centro di Ricerca, Sperimentazione e Formazione in Agricoltura Basile-Caramia (CRSFA), via Cisternino, 281 Locorotondo, Bari, Italy; 4 Istituto per la Protezione Sostenibile delle Piante (CNR-IPSP), via Amendola 122/D, Bari, Italy; 5 CINECA, SCAI Super Computing Applications and Innovation, via dei Tizii 6, Rome, Italy; 6 Phytosanitary Service, Regione Emilia-Romagna, Bologna, Italy; 7 CREA, Research Centre for Olive, Citrus and Tree Fruit, via La Canapona 1 bis, Forlì, Italy

**Keywords:** Genome-wide association, Sharka disease, PPV tolerance, Peach, *Prunus*

## Abstract

**Background:**

*Plum pox virus* (PPV), agent of Sharka disease, is the most important quarantine pathogen of peach (*P. persica* L. Batsch). Extensive evaluation of peach germplasm has highlighted the lack of resistant sources, while suggesting the presence of a quantitative disease resistance, expressed as reduction in the intensity of symptoms. Unravelling the genetic architecture of peach response to PPV infection is essential for pyramiding resistant genes and for developing more tolerant varieties. For this purpose, a genome-wide association (GWA) approach was applied in a panel of accessions phenotyped for virus susceptibility and genotyped with the IPSC peach 9 K SNP Array, and coupled with an high-coverage resequencing of the tolerant accession ‘Kamarat’.

**Results:**

Genome-wide association identified three highly significant associated loci on chromosome 2 and 3, accounting for most of the reduction in PPV-M susceptibility within the analysed peach population. The exploration of associated intervals through whole-genome comparison of the tolerant accession ‘Kamarat’ and other susceptible accessions, including the PPV-resistant wild-related species *P. davidiana*, allow the identification of allelic variants in promising candidate genes, including an *RTM2-like* gene already characterized in *A. thaliana*.

**Conclusions:**

The present study is the first effort to identify genetic factors involved in Sharka disease in peach germplasm through a GWA approach. We provide evidence of the presence of quantitative resistant loci in a collection of peach accessions, identifying major loci and highly informative SNPs that could be useful for marker assisted selection. These results could serve as reference bases for future research aimed at the comprehension of genetic mechanism regulating the complex peach-PPV interaction.

**Electronic supplementary material:**

The online version of this article (10.1186/s12870-017-1117-0) contains supplementary material, which is available to authorized users.

## Background

*Plum pox virus* (PPV) gen. *Potyvirus*, agent of Sharka disease, is the most devastating viral pathogen of stone fruits, particularly peach (*P. persica* L. Batsch). Since the first report of PPV infections in Bulgaria [1], the virus has gradually spread worldwide. The control of virus spread is currently based on a series of preventive measures, such as outbreaks monitoring and eradication of affected plants. Nevertheless, these strategies have barely slowed spread of the virus, which is by now endemic in many European growing areas [2]. Among several PPV strains identified so far (D, M, Rec, EA, C, W, and T), the M isolate is by far the most virulent in peach [3].

The identification and exploitation of PPV-resistant sources represents the main eligible strategy for the long-term protection of peach cultivation. Genetic sources of both PPV-M and -D resistance have been identified and characterized in apricot (*P. armeniaca* L.) through a series of linkage and association studies, allowing the development of molecular markers and breeding programmes for the transfer of resistance to novel varieties [4]. In contrast, immunity or resistance against PPV-M have not yet been reported in peach, but only in the related species *P. davidiana* and *P. dulcis* (almond) [5, 6]*.* The introgression of resistance through hybrid selections has been unsuccessful so far due to several drawbacks, including the lack of resistance-associated molecular markers. The vast majority of peach accessions are highly susceptible to the PPV-M strain, although genotypes showing lesser symptom severity upon infection have been reported [7, 8]. Most, if not all, the peach varieties are symptomless when infected by PPV-D strain [9]. The Italian PPVCON research project [10] enabled a wide evaluation of several peach germplasm collections, allowing the identification of a few tolerant accessions, characterized by virus replication and spread throughout graft-infected plants but essentially symptomless or developing only sporadic, mild symptoms. This tolerance may represent an important trait for the short-term preservation of peach cultivation in endemic areas, although there are conflicting views among virologists about the possible impact of cultivating tolerant plants [11].

The range of phenotypic response to virus infection observed in peach germplasm suggests the existence of a quantitative disease tolerance, able to confer a reduction in symptoms intensity rather than absence of the disease. The identification of genetic loci controlling such quantitative tolerance may allow the design of molecular markers to assist breeders in pyramiding favourable alleles and the development of tolerant varieties. However, the genetic architecture of PPV-M tolerance is still unknown in peach and only limited information is available about the resistance alleles present in related species. Linkage mapping experiments in ‘Summergrand’ (peach, susceptible) x ‘clone P1908’ (*P. davidiana*, resistant) progenies suggest a complex pattern of polygenic inheritance [12, 13]. Minor quantitative resistance loci (QRLs) have been identified on LG (linkage group) 1, 2, 4, 6 and 7. However, the same QRLs were only partially confirmed in a cross ‘Rubira’ (peach) x ‘P1908’, suggesting that their position, number and effect vary depending from the genetic background of peach parents [14]. The small number of molecular markers and progeny sizes adopted in such mapping experiments made it difficult to identify the genomic regions and putative candidate gene(s) associated to the trait. In apricot, a major determinant for PPV resistance -most probably corresponding to a *MATH* gene [15] belonging to the *TRAF*-like gene family- has been fine-mapped at the *PPVRes* locus on LG1, although its effect also varies depending on the genetic background [16]. In the model species *Arabidopsis thaliana*, dominant resistance against PPV is also conferred by a *MATH* gene [17, 18], suggesting functional conservation across plant species. A major mechanism conferring resistance against potyviruses involving eukaryotic translation initiation factor (eIF) proteins has also been demonstrated in several model and non-model species [19]. Silencing of the *eIF(iso)4E* gene confers PPV resistance in plum [20] and peach [21]. However, low genetic diversity in *eIF* genes seems to be present within peach germplasm (Decroocq, unpublished results).

In peach biparental linkage mapping approaches often offer limited genetic resolution, due to small progeny size and lack of polymorphisms in genomic regions identical-by-descent. Particularly for traits influenced by the genetic background, as virus resistance, it is difficult to achieve an overall picture of involved loci using a limited number of parents. The genome-wide association (GWA) approach is becoming an increasingly powerful tool to identify loci controlling both quantitative and qualitative traits, bearing the potential to improve the power of detection in comparison with classical biparental linkage mapping [22]. This approach relies on historical recombination events occurred in natural populations and collections of landraces, breeding materials and varieties, establishing marker-trait associations based on linkage disequilibrium (LD), the non-random association of alleles at two or more loci [23]. The effectiveness of GWA largely depends on LD extent and distribution, which in turn are affected by biological and evolutionary factors. The presence of population structure and familial relatedness, i.e. systematic difference in allele frequencies between subpopulations, is particularly problematic for GWA since it leads to spurious associations and increase in false positives [24]. As a consequence of self-mating system, peach is characterized by high levels of inbreeding [25]. Moreover, peach germplasm has undergone a series of bottlenecks due to domestication-related events and modern breeding activities, started in the middle of the twentieth century from a low number of parents [26]. These phenomena along with artificial selection contributed to reduce genetic diversity in cultivated peach, increasing LD levels compared to wild relatives and generating different sub-populations [27–30]. Recent examples of the use of GWA approaches in peach showed promising results for the mapping of both quantitative and qualitative traits ([31] [32]). In the present work, a GWA approach was applied in a panel of peach accessions, genotyped with 9 K SNP Array to identify genomic regions associated to Sharka disease tolerance and putative markers to be used for assisted breeding.

## Results

### Phenotyping results

**Fig. 1:**
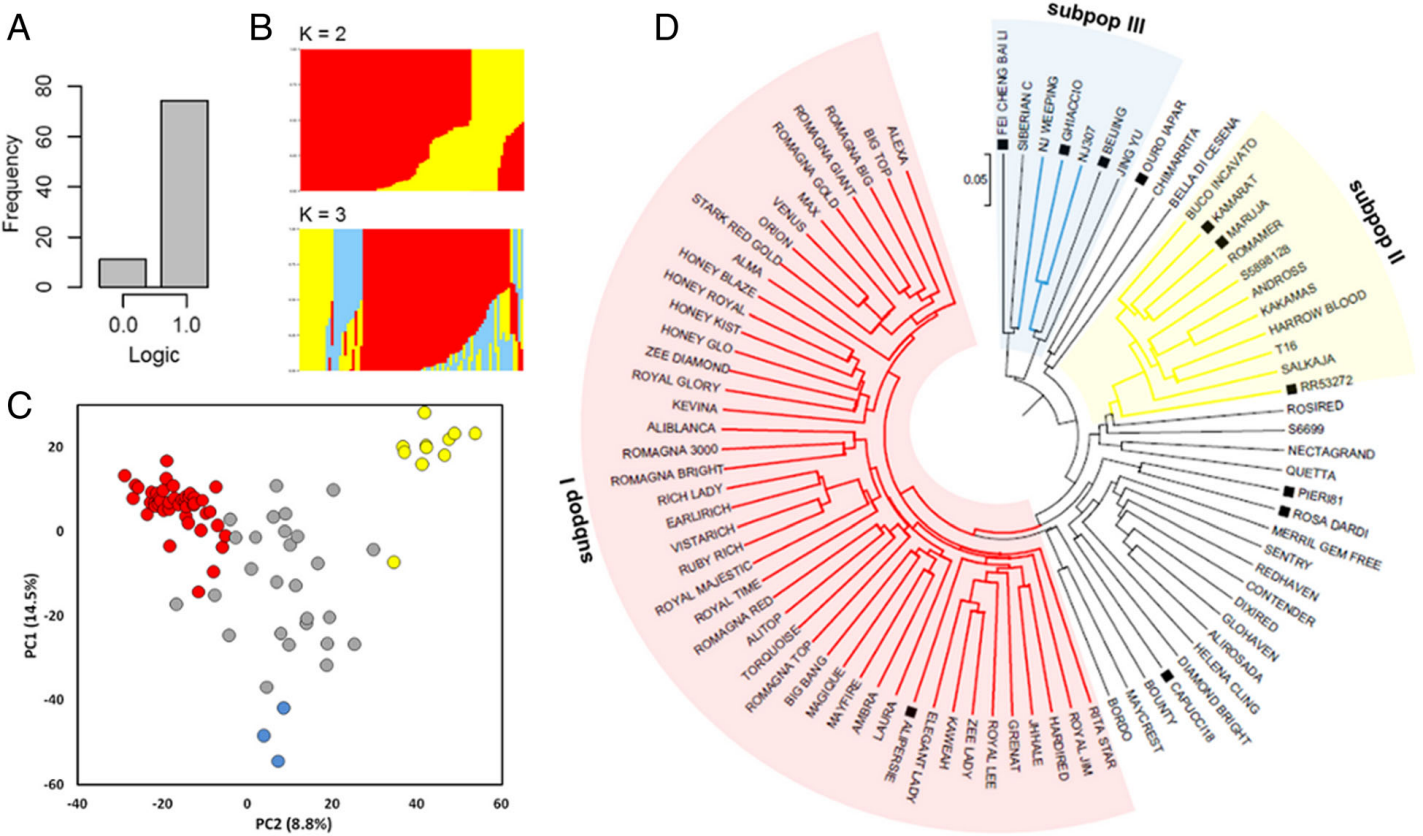
**a** Histogram summarizing the frequency of tolerant (0) vs susceptible (1) phenotypes in the panel of 85 accessions used for GWA analysis; **b** Genetic structure plot of the analyzed panel for the optimal number of a priori genetic clusters (K = 2 and K = 3), with ancestry proportion on the Y-axis. The red, yellow and light blue bars indicate the subpopulations I (breeding-derived), subpopulation II (Occidental non-breeding) and subpopulation III (Oriental origins), respectively; **c** PCA scatter plot of the first two principal component (PC1 and PC2) of the genetic relationship matrix for the analyzed panel. Red, yellow and light blue colours indicate the three subpopulation (I, II, III), respectively. The proportion of the explained variance is indicated in parenthesis; **d** Unrooted UPGMA tree showing the phylogenetic relationship among accessions. Black square indicate tolerant accessions. Red, yellow and light blue halos mark the assignment of each taxa to the respective subpopulations with a membership probability >0.8

Evaluation trials have shown an overall high susceptibility of peach germplasm and the absence of resistant accessions (no symptoms, RT-PCR negative) against the PPV-M strain infection. Based on visual assessment (score from 0 to 3), most accessions showed moderate to severe symptoms on leaves (class 2 and 3, respectively) since the first year after inoculum, whereas others became symptomatic only at the third or fourth year (Additional file 1). The intensity of symptoms stabilized generally from the second year, while maintaining a certain variability for some accessions. Compared to leaves, fruits were less sensitive and in some cases they were symptom-less even in accessions with severe foliar damage. Nevertheless, the screen house conditions did not allow the evaluation of fruits for the whole panel. A restricted number of accessions were classified as tolerant, because they were asymptomatic or displayed sporadic and/or mild (class 1) symptoms, while testing positive to the ELISA and/or RT-PCR assays. In particular, ‘Ghiaccio1’, ‘Kamarat’, ‘Maruja’, ‘Ouro Iapar’, ‘Pieri81’ and ‘RR53–272’ were asymptomatic during screen house trials, whereas ‘Capucci18’, ‘Bei Jing’ and ‘Alipersié’ showed mild and sporadic symptoms, even more severe in ‘Fei Cheng Bai Li’, accompanied by a certain recovery ability. A field trial in an endemic area heavily infested by PPV-M strain (Verona, Italy) confirmed the high degree of tolerance for ‘Ghiaccio1’, showing no symptoms on flower, leaf and fruit after four years of evaluation. On-field trials also confirmed the low susceptibility of ‘Rosa Dardi’, a tolerant accession previously reported by Casati et al. [33] and some other accessions previously classified as tolerant, although symptoms tended to be slightly more pronounced compared to those observed in controlled conditions (Additional file 1). For GWA analysis, phenotypic data were coded as binary phenotype (tolerant vs susceptible) (Additional file 2 and Fig. 1a).

### Population structure

Population stratification in the analyzed accessions was inferred in ADMIXTURE. According to a previous classification [32] the cluster of breeding-derived accessions (subpop I) was clearly differentiated from Occidental non-breeding ones (subpop II) for K = 2, explaining most of the ancestry within the panel (Fig. 1b). For K = 3, additional groups of admixed individuals were separated, particularly a small cluster of individuals with prevalent Oriental origins (subpop III) (Fig. 1b). Most of the admixture was shared between subpop I and II, whereas only three individuals were assigned to subpop III with a membership probability of *p* > 0.8. Such stratification is also captured by the principal component analysis (PCA) (Fig. 1c). The separation of three clusters was not absolute and a discrete number of accessions occupied a centric position. PC1 and PC2 captured 14.5% and 8.8% of the total explained variance, respectively. Stratification patterns were in agreement with UPGMA hierarchical clustering, supporting the dendrogram morphology (Fig. 1d). Tolerant genotypes were not equally distributed among the identified subpopulations, and mainly belonged to the Occidental non-breeding cluster and the small group with Oriental ancestry. No tolerant individuals were found within the Occidental breeding-derived group. As a consequence, phenotypes tend to correlate with population structure, as explained by the first two PCs (Additional file 3: Fig. S1). An approximate estimation in the analyzed population suggests a slow LD decay with the physical distance between markers, with an overall r^2^ value dropping below 0.2 at about 0.85 Mbp (Additional file 4: Fig. S2).

### GWA analysis

As a proof-of-concept of the statistical power of GWA approach in the considered panel, we analyzed two already characterized Mendelian traits, fruit flesh colour controlled by locus *Y* (white/yellow, *Y/y*) and skin pubescence controlled by locus *G* (peach/nectarine, *G/g*). Based on previous knowledge about the position of causal mutations for both traits [34, 35], the best accuracy was achieved using the FarmCPU algorithm adjusted for population structure (Q matrix for K = 3) (Additional file 5: Fig. S3). Despite the small panel size, the distance of most associated SNP markers from true positions of the loci resulted substantially improved for both traits, as compared to a previous reported association analysis [32] (Additional file 5: Fig. S3). These results support the validity of our panel for GWAS and the high statistical power of FarmCPU algorithm.

**Fig. 2:**
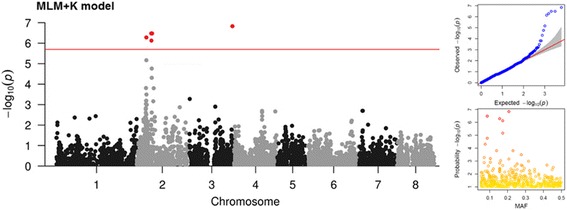
Manhattan plot of the -log_10_ *p*-values estimated for binary (tolerant vs susceptible) coded phenotypic response to PPV-M infection in the panel of 85 accessions using standard Mixed Linear Model algorithm adjusted for kinship (MLM + K model). Red circle indicates significant SNPs passing the Bonferroni-adjusted threshold (red horizontal line) based on the effective number of independent tests (−log_10_ 2e-06). Quantile-quantile (QQ)-plot (top right panel) and minor allele frequencies (MAF) vs -log_10_ *p*-values (bottom right panel) are also shown

Considering the small panel size and the complexity of the dataset (low frequency of tolerant individuals), different statistical models were tested for detecting associations for PPV tolerance. As expected from the presence of population stratification, strongly inflated *p*-values were observed when using naive GLM model (data not shown). The inclusion as covariates of either the first two PCs or Q matrix (for K = 3) tend to ameliorate *p* distribution, although a relevant number of false positives was still present (Additional file 6: Fig. S4). The application of MLM models allows to better account for stratification, reducing false positive associations and increasing the statistical power (Fig. 2). MLM + K model showed a good fit for *p*-values, irrespective of the algorithm used for calculating the kinship matrix (Additional file 7: Fig. S5). Significant SNPs associated to PPV tolerance were distributed over chromosome 2 and 3 (Table 1). The strongest signal, consisting of a single SNP (SNP_IGA_366639) with a *p*-value of 1.49e-07, was detected at about 26 Mb on chromosome 3. Five significant markers, comprised between SNP_IGA_214703 and SNP_IGA_218596 (3.32e-07), span a 400 Kb genomic region located at about 9 Mb on chromosome 2. Another locus was identified at about 5.7 Mb on the same chromosome (SNPs, SNP_IGA_185608 and SNP_IGA_185721). Similar results were obtained by testing the compressed (CMLM + K) model (Additional file 8: Fig. S6A).

**Table 1:** SNPs associated with PPV-M tolerance in a panel of 85 accessions. Chromosome positions, minor allele frequencies (MAF), *p* values of Hardy-Weinberg equilibrium (HWE) and respective *p* values under different GWA models

Array ID#	Chr	Pos	MAF	HWE	MLM + K	CMLM + K	SUPER	FarmCPU
				*p* - value				
SNP_ IGA_185608	2	5,772,285	0.15	0.10	5.23e-07	1.65e-05	1.91e-07	–
SNP_ IGA_185721	2	5,778,677	0.17	0.06	6.79e-06	1.68e-05	1.80e-05	–
SNP_ IGA_214703	2	8,639,144	0.09	0.12	3.32e-07	6.86e-07	3.29-e09	3.11e-12
SNP_ IGA_215563	2	8,748,723	0.17	1.00	7.46e-07	–	–	–
SNP_ IGA_217815	2	8,955,153	0.09	0.16	1.73e-05	–	–	–
SNP_ IGA_217794	2	8,950,628	0.07	0.16	4.89e-05	–	–	–
SNP_ IGA_218596	2	9,031,544	0.09	0.12	3.32e-07	7.77e-05	–	–
SNP_ IGA_258078	2	16,998,043	0.08	0.14	–	–	–	6.27e-09
SNP_ IGA_366639	3	26,378,711	0.21	0.94	1.49e-07	9.09e-06	1.09e-06	4.82e-07

**Fig. 3:**
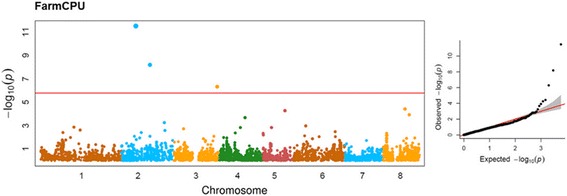
Manhattan plot (left panel) and QQ-plots (right panel) of -log_10_ *p*-values estimated for binary (tolerant vs susceptible) coded phenotypic response to PPV-M infection in the panel of 85 accessions using FarmCPU algorithm adjusted for population structure (Q-matrix calculated for K = 3) (left panel). Red circle indicates significant SNPs passing the Bonferroni-adjusted threshold (red horizontal line) based on the effective number of independent tests (−log_10_ 2e-06)

The addition of covariates tend to generate over-fitted models, as deduced by the deflated *p*-values of respective QQ-plot (Additional file 8: Fig. S6B). This suggests that the kinship is suitable to capture most of the genetic relationships within the panel. Nonetheless, the small cluster of individuals composing subpop III appears not sufficiently captured by the kinship matrix. Considering that 4 out of 11 tolerant individuals derived from this cluster, we further tried to include population structure effects by applying the SUPER model: this model derives individuals kinship from a subset of pseudo-QTNs after excluding those in LD with the tested SNPs and is particularly useful when covariates tend to mask associations. The model improved the resolution of previously identified signals, although with higher background inflation (Additional file 8: Fig. S6C). Finally, the application of FarmCPU algorithm further confirm the association of SNP_IGA_366639 and refined the multiple signals detected on chromosome 2, suggesting a stronger association for SNP_IGA_214703. FarmCPU detected also an additional locus, SNP_IGA_258078 (Fig. 3).

### Model selection and validation

For an approximate estimation of effect size, a logistic regression was fitted at significant SNPs on binary phenotypes. As observed by odds ratio under the assumption of a dominant genetic model, highly significant associations were confirmed for SNP_IGA_366639 (OR = 43.07), SNP_IGA_214703 (OR = 23.53) and SNP_IGA_185608 (OR = 20.66), although with quite large confidence intervals (Table 2). For all three loci, the minor allele was associated with a decrease in PPV-M susceptibility (i.e. increased tolerance). The three SNPs were also prioritized by LASSO penalized regression approach, although only SNP_IGA_366639 appeared statistically significant (Table 2). Potential interactions among loci were also explored through MDR analysis. Among the predicted models, the best interaction includes the combination of SNP_IGA_366639 and SNP_IGA_185608, with a testing accuracy of 82.3% and consistency of 9/10, statistically significant for *p* ≤ 0.05, as determined empirically by permutation testing (data not shown).

**Table 2:** Allelic effect estimation by standard and penalized (LASSO) logistic regression using associated SNPs loci. Odds ratio, p-values, confidence intervals and the genetic model for standard logistic regression are reported

SNP	Alleles	Logistic Regression			LASSO		
		OR	*p* value	L95 - U95	Model	beta	*p* value
SNP _IGA_185608	**A**:C	20.66	0.00012	4.41–96.67	DOM	0.382	0.276
SNP_ IGA_185721	**C**:A	11.47	0.00011	3.32–39.61	–	–	–
SNP_ IGA_214703	**G**:A	23.53	8.67e-05	4.86–113.9	DOM	0.145	0.117
SNP_ IGA_215563	**A**:C	16.56	0.00023	3.71–73.78	DOM	–	–
SNP_ IGA_217794	**G**:T	9.30	0.00137	2.37–36.46	DOM	–	–
SNP_ IGA_217815	**A**:C	9.30	0.00137	2.37–36.46	DOM	–	–
SNP_ IGA_218596	**C**:T	11.10	0.00121	2.58–47.72	DOM	–	–
SNP_ IGA_258078	**G**:A	5.47	0.00028	2.18–13.74	ADD	–	–
SNP_ IGA_366639	**A** **:**C	43.07	1.77e-05	7.72–240.2	–	0.334	2.0e-06

Alleles associated with PPV-tolerance are in bold and underlined

**Fig. 4:**
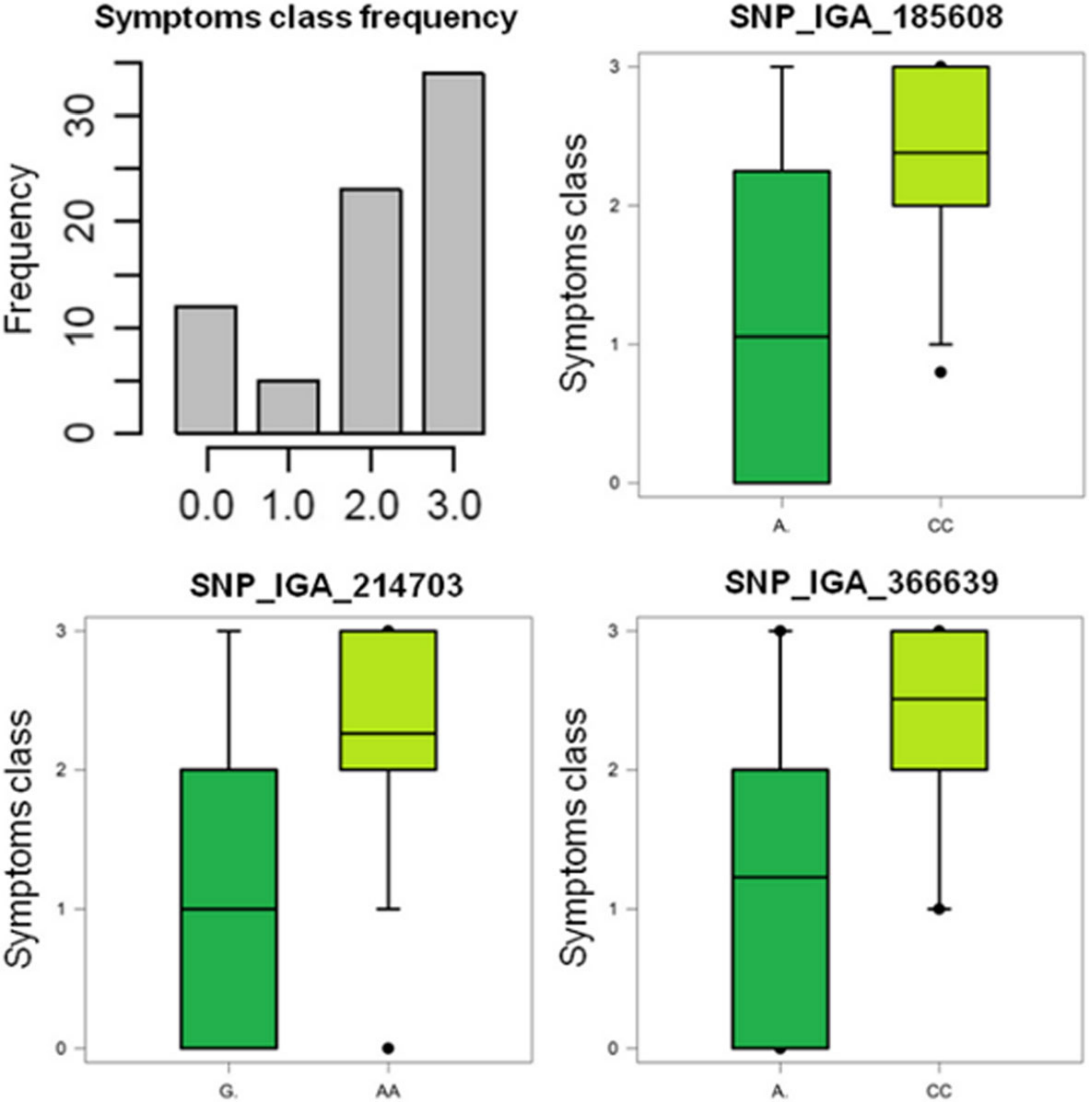
Box-plots of markers-trait association for the three SNPs detected by GWA analysis (SNP_IGA_185608, SNP_IGA_214703 and SNP_IGA_366639) with symptoms intensity classes as inferred by the non-parametric Kruskal-Wallis K-test (*p* < 0.01) in a panel of 73 accessions. The frequency of the four classes of symptoms severity (asymptomatic, 0, to severe, 3) is shown in the top right panel

**Fig. 5:**
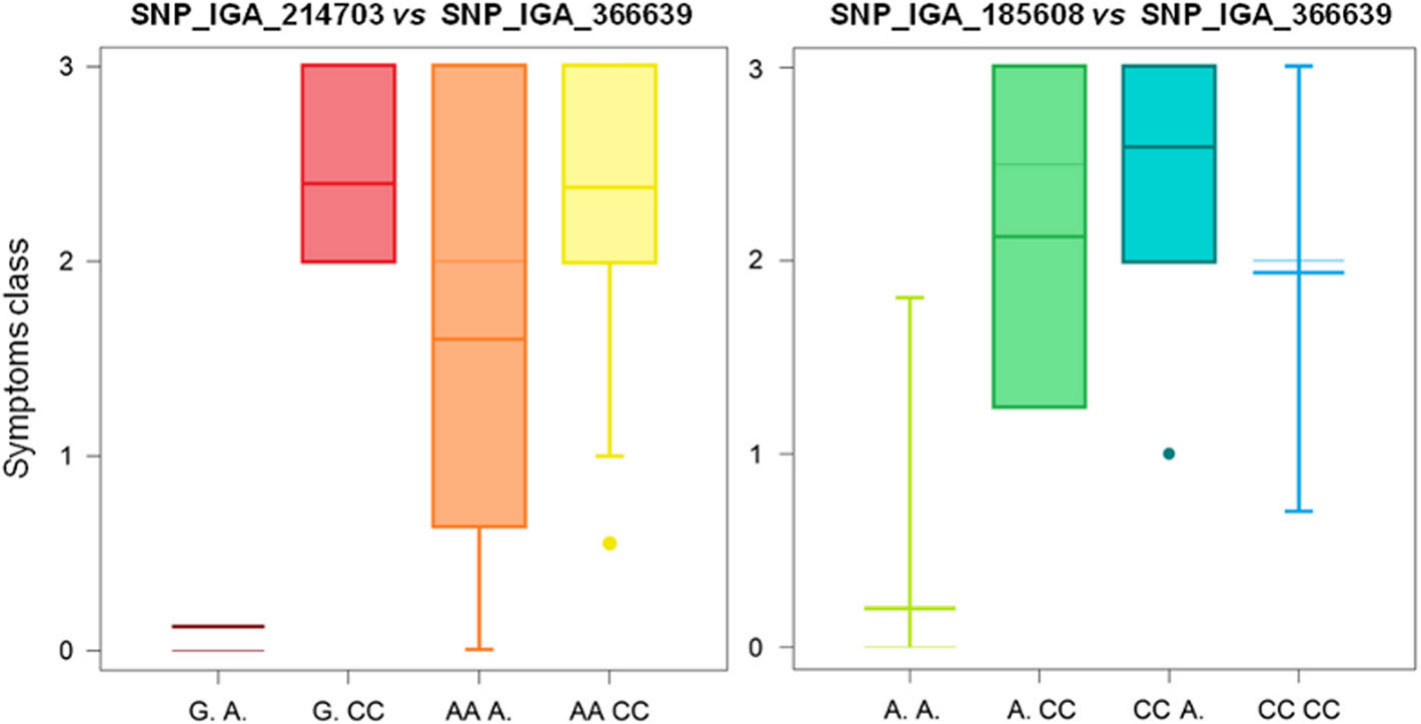
Box-plots of markers pairwise effects on symptoms intensity (0–3 scale) as inferred by the non-parametric Kruskal-Wallis K-test (*p* < 0.01) in a panel of 73 accessions. In the left and right panels, the locus-locus interactions involving SNP_IGA_214703 (G/A) vs SNP_IGA_366639 (A/C) and SNP_IGA_185608 (A/C) vs SNP_IGA_366639 (A/C) are shown, respectively

The association of the three SNPs to reduced symptom intensity was further confirmed using 0–3 classes of symptoms intensity in 73 accessions (25 not present in the GWA panel) (Additional file 1; Fig. 4). In addition, pairwise comparisons among the three loci suggest a non-linear additive interaction: SNP_IGA_366639 appears necessary for expressing a high degree of tolerance (class 0) with a synergic but apparently redundant effect of either SNP_IGA_214703 or SNP_IGA_185608 (Fig. 5).

### Prediction of candidate genes for PPV-M tolerance

**Fig. 6:**
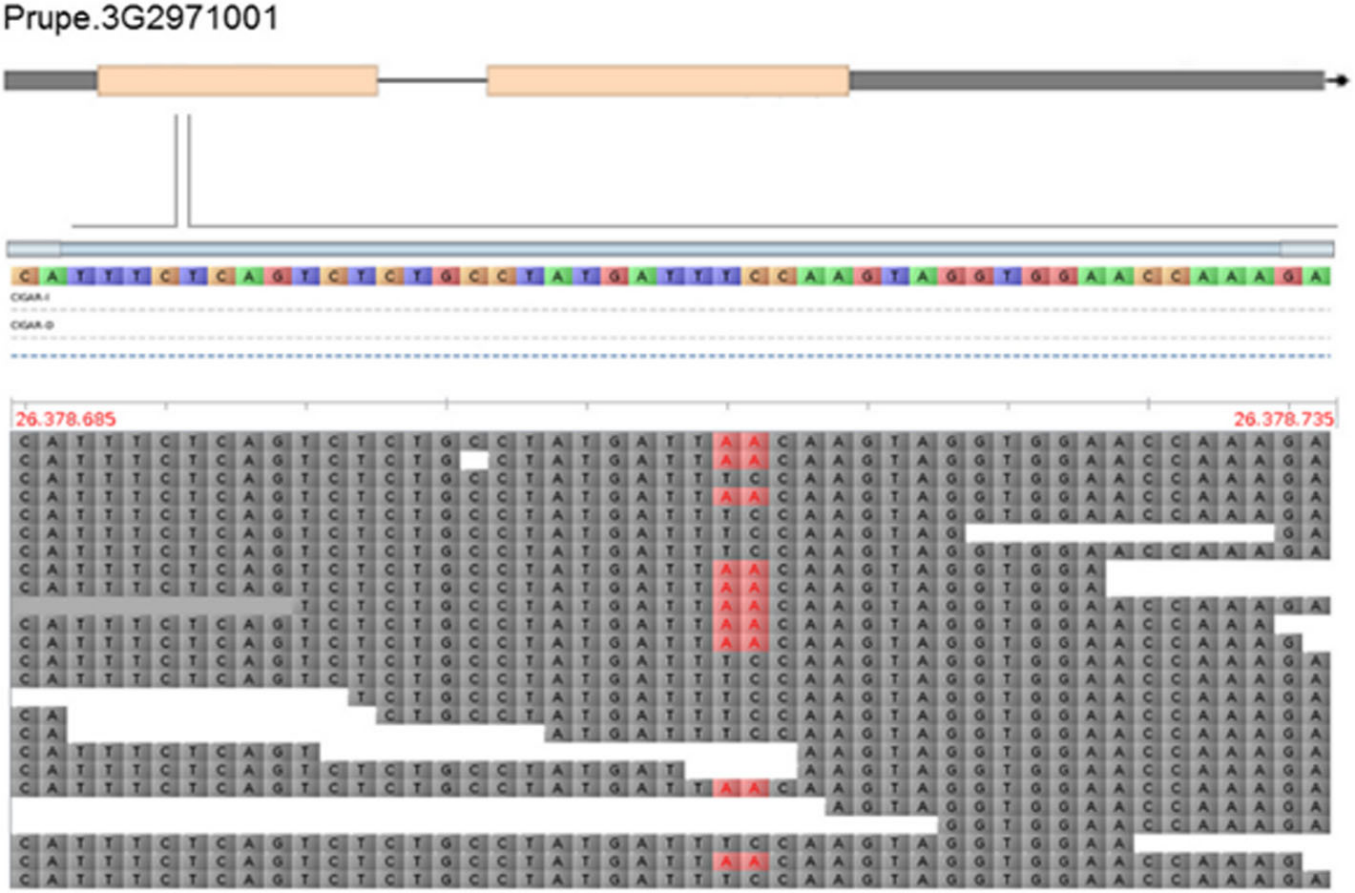
Allelic variant identified in the exon I of the Prupe.3G2971001 gene (coding for an ENODL-like protein) from whole-genome sequencing data of ‘Kamarat’ accession, as visualized in Tablet software

**Fig. 7:**
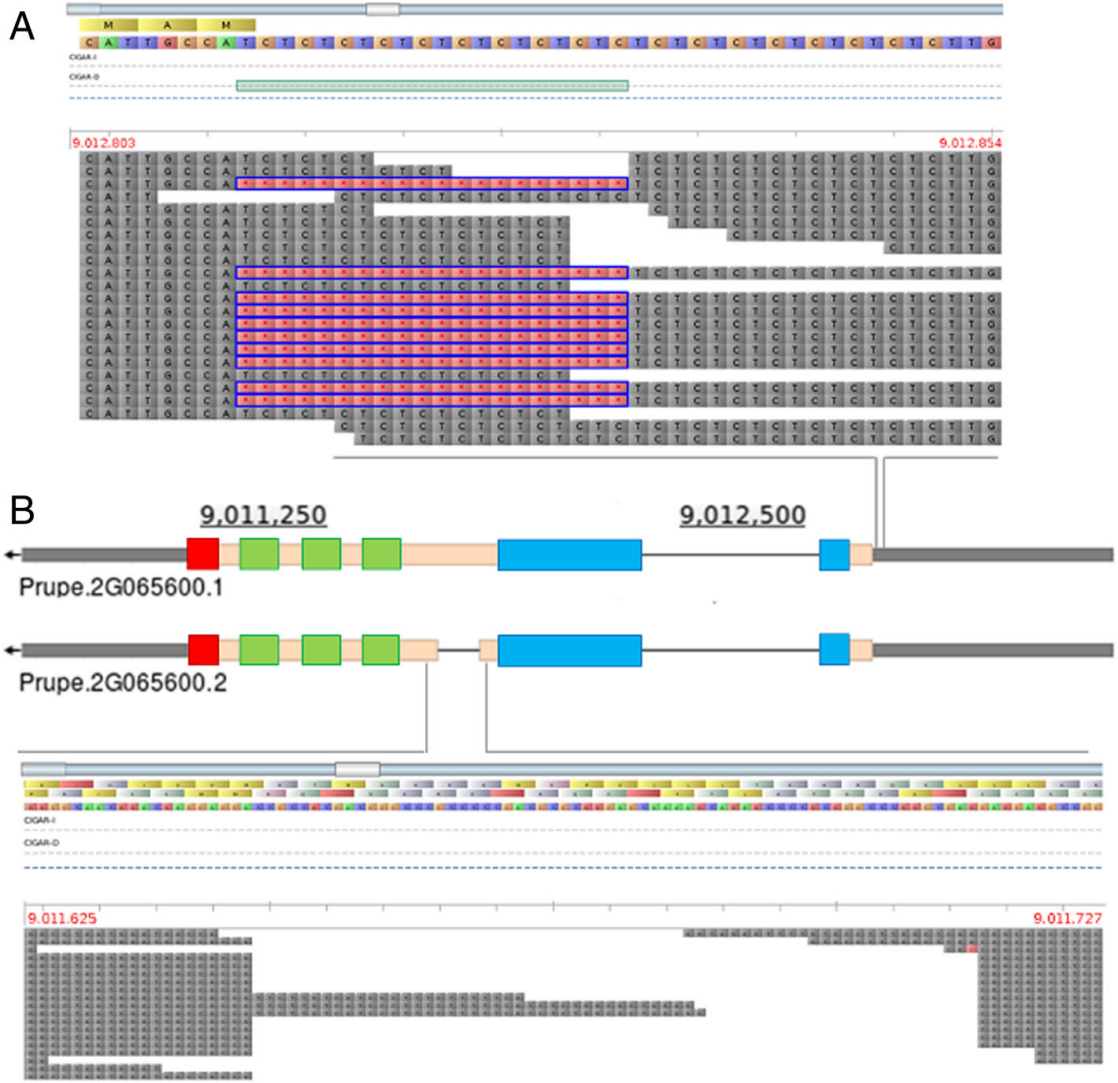
Allelic variants identified on the Prupe.2G065600 gene (coding for an RTM2-like protein) from whole-genome sequencing data of ‘Yumyeong’ accession, as visualized in Tablet software. **a** Microsatellite (CT/GA-repeat) deletion within the 5′-UTR region; **b** 63 nucleotide deletion within the exon II. Position of the conserved α-crystalline-like domain, the coiled-coils regions (predicted by COILS software) and C-terminus transmembrane domain (predicted by TMHMM) are indicated by blue, green and red boxes, respectively

Candidate genes for PPV-M tolerance/susceptibility were searched within genomic regions around the most associated SNPs, based on detailed annotation for ‘Lovell’ peach reference genome (Additional file 9). To account for possible ascertainment bias in estimating LD pattern from SNP array data, the identified regions were extended at least of 200 Kb on both sides of the associated intervals. Further insights into sequence variants possibly associated with PPV response were obtained by re-sequencing data of tolerant and susceptible accessions, including the resistant *P. davidiana* ‘P1908’. SNP_IGA_366639 identified on chromosome 3 falls within the coding region of an *ENODL-like* gene (Prupe.3G291466) belonging to Nodulin-like protein family. WGS data of ‘Kamarat’ and ‘Yumyeong’ showed the presence of an additional A/T polymorphism adjacent to the A/C one tagged by the array, generating two allelic variants AA/TC, respectively (Fig. 6). The tolerance-associated AA variant introduces a premature stop codon in the predicted open reading frame. Apart from this finding, the region is characterized by high LD level and gene density, but low genetic diversity (Additional file 10: Fig. S7 and Additional file 11: Fig. S8). The candidate list includes other genes with no apparently relevant mutations in tolerant/resistant genotypes (Additional file 12: Table S1). In contrast, the region on chromosome 2 delimited by SNP_214703 and SNP_218596, is characterized by a low gene density but high genetic diversity (Additional file 10: Fig. S7 and Additional file 13: Fig. S9). The SNPs tagged by the array are all synonymous mutations, with no clear impact on gene function. Exploring CGs present in the interval, the Prupe.2G065600 gene, encoding an RTM2-like protein related to a protein involved in the restriction of *Potyvirus* movement in Arabidopsis [36], was identified. In ‘Kamarat’ and ‘Yumyeong’ (seed parent of ‘Ghiaccio1’) a heterozygous allele with a partially truncated repeat within the 5′ UTR was found, while this variant is homozygous in the resistant *P. davidiana* (Fig. 7a). Only in ‘Yumyeong’, this allelic variant also shows a 63 nucleotides deletion in the exon II (Fig. 7b). Other candidate genes include a DEA(D/H)-box RNA helicase, with several aminoacidic substitutions, genes encoding a cycling DOF factor (CDF2) and SKP1/ASK1 protein, both showing two putative loss-of-function mutations in ‘Kamarat’ (Additional file 14: Table S2). The two SNPs located at about 5.7 Mb on chromosome 2 fall within the coding region of a MYB33/65-like (Prupe.2G050100) and a tyrosine kinase (Prupe.2G050000), respectively, in low LD with the surrounding regions (Additional file 15: Figure S10). Among the other predicted genes within the region, none was apparently related to plant-virus interactions (Additional file 16: Table S3).

### Validation of markers and candidate genes

**Fig. 8:**
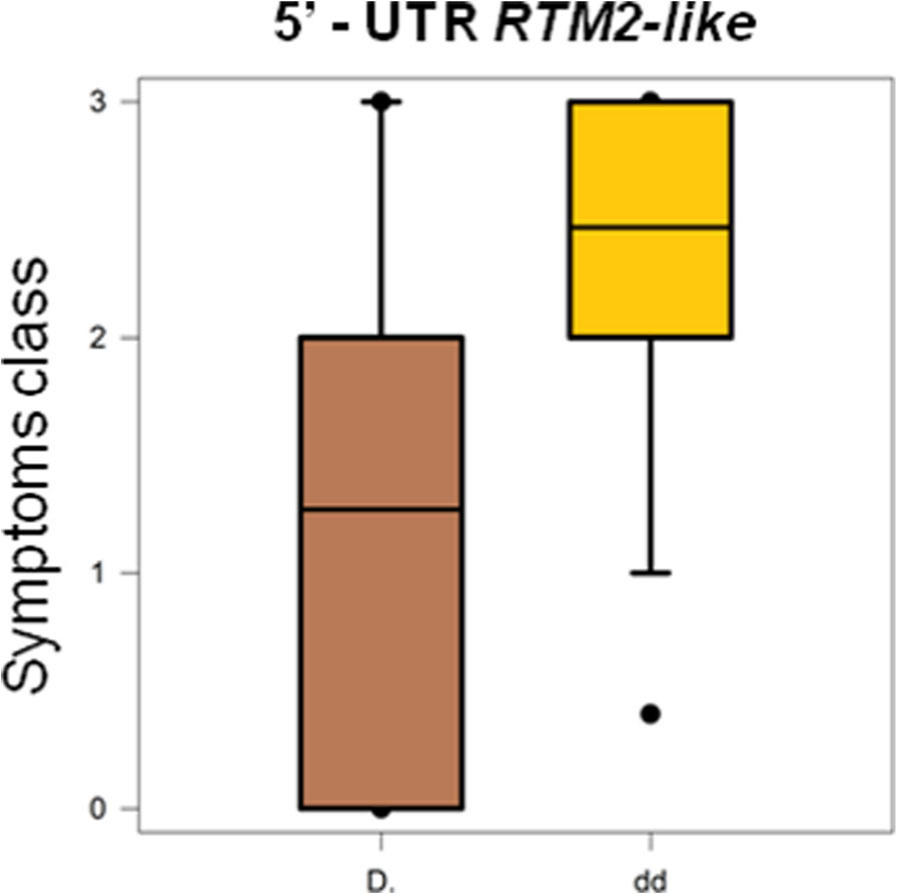
Box-plots of the association between the 5′-UTR variant on RTM2-like gene Prupe.2G065600) with the expression of symptoms intensity as inferred by the non-parametric Kruskal-Wallis K-test (*p* < 0.01) in a panel of 73 peach accessions scored using four classes of symptoms severity (asymptomatic, 0, to severe,3)

A significant association for the *RTM2-like* 5′-UTR variant with reduced disease severity was confirmed in the panel of 73 accessions (Fig. 8). The variant is present in all tolerant accessions, excluding ‘Fei Cheng Bai Li’. The involvement of the 63 nt deletion on exon II resulted less clear, since the variant is absent in the tolerant accession ‘Kamarat’ and the resistant *P. davidiana* ‘P1908’ (Additional file 9). Both *RTM2-like* gene variants were further evaluated in three pseudo BC1 progenies ‘Orion’ x ‘SD’ (Summergrand x *P. davidiana* ‘P1908’), segregating for the partial CT/GA-repeats deletion in the 5′-UTR (parents are all heterozygous for the mutation) and for the 63 nt deletion (heterozygous in ‘Orion’ and absent in SDs) with an expected ratio of 1:2:1 and 1:1, respectively. Four out 70 individuals were classified as resistant (no symptoms, RT-PCR negative), 10 as tolerant (no symptoms, RT-PCR positive) and the remaining 56 as susceptible (Additional file 17). At least in this hybrid genetic background, single marker analyses showed no significant co-segregation between 5′-UTR variant and symptom intensity nor PPV-M tolerance/resistance, and only slight effect of the exon II deletion (Additional file 18: Fig. S11).

## Discussion

Currently, Sharka disease is one of the most important phytosanitary issues in peach. The absence of intraspecific source of resistance and the complexity of introgression from related species has prompted the search of alternative approaches, including genetic engineering [11].

In agreement with several other studies, we confirmed the high susceptibility of peach to PPV. Almost all breeding-derived accessions were rapidly infected by the virus, developing moderate to severe symptoms. A restricted number of accessions clearly showed different responses against viral inoculum in screen house conditions, including delayed disease appearance, mild or no visible symptoms and a certain recovery ability. These accessions mostly belong to the ‘Occidental traditional’ cluster or are ‘admixed’ genotypes with prevalent Oriental ancestry. The tolerance of these accessions was also confirmed by field trials under very high inoculum pressure, although symptoms were slightly more severe, as previously reported for many resistant lines derived from *P. davidiana* hybrids [37].

The plant response to virus infection is a complex trait, affected by several factors including viral strains and environmental conditions [38]. A common concern in evaluating peach response to PPV infection is the lack of an objective method to measure and compare responses among genotypes. Symptoms evaluation through visual inspection and attribution of score classes are affected by a certain degree of subjectivity, which hampers accurate differentiation of the specific response in each accession. Clearly, the quality of the phenotypic data has significant bearing on the accuracy of GWA, particularly for differentiating a quantitative disease response [39, 40]. The classification of plants behaviour as binary outcome (tolerant vs susceptible) has been proven to be useful in apricot, at least for detecting loci with major effects [41–43]. Anyway, the search for more objective phenotyping methods is a current research priority, as well as a more stringent and reliable evaluation of the plants through experiments in natural conditions.

In this work we provide a first insight into quantitative resistance loci affecting Sharka disease tolerance in peach by using an association mapping approach. A recent study in apricot has demonstrated the power of GWA in detecting both known and novel PPV resistance loci, even in a small size population [42]. GWA requires a genomic map in which marker density is higher than the LD extent [44]. The moderate to high LD levels observed in peach and the effective marker density deployed in this study (about 1 marker every 40 Kbp), appear sufficient to tag associated loci. This is maybe particularly true for traits under biotic selection, often underpinned by a small number of large-effect loci [45].

In terms of accuracy, the effectiveness of the GWA approach is largely determined by the level of population stratification. Although a small panel size may introduce bias in the estimation of genetic relationships, in our case the structure of the analysed population and individual membership reflect those observed in another study comprising more than 1500 peach accessions [32]. Accounting for the complexity of the phenotypic dataset and the known stratification of our peach population, different algorithms were tested for modeling marker-trait associations. The Mixed Linear Model (MLM) outperforms the prediction of both naïve and structured GLM, since it better accounts for genetic relationships among individuals. The different MLM algorithms tested provide substantially similar results in terms of identified loci and their significance. FarmCPU seems to improve statistical power and resolution of GWA analysis, as also demonstrated by detection of already validated loci controlling fruit flesh colour and fruit pubescence. By applying stringent thresholds for reporting significant associations and different models to control for population structure and relatedness, clear signals were identified on chromosomes 2 and 3. In support of these main associations, they were recovered in all tested models. This is the first report about the presence of genetic factors regulating Sharka disease susceptibility in peach and, therefore, we can only compare results with those reported from interspecific peach cross with *P. davidiana* or in other species of the *Prunus* genus. The presence of quantitative resistance loci on chromosome 2 was previously observed by linkage mapping in both ‘Summergrand’ x ‘P1908’ and ‘Rubira’ x ‘P1908’ hybrid progenies [12, 14]. In contrast, the locus identified on chromosome 3 has not been reported in such experiments, although weak associations in a collinear region seem to be present in apricot [42]. Logistic regression and MDR analyses on binary phenotypes, and pairwise comparisons on classes of symptoms intensity in a panel of accessions, suggested that SNP_IGA_366639 and either SNP_IGA_214703 or SNP_IGA_185608, sufficiently account for the quantitative reduction of disease severity. However, their effectiveness also varied depending on the specific genotype background, suggesting the presence of additional genetic factors with epistatic and/or minor additive effects. Considering the small panel size and number of SNP markers, it is likely that only major effect loci were detected in our GWAS.

The estimation of linkage disequilibrium (LD) patterns is critical for mapping resolution and the definition of the window size around the significant SNPs [46, 47]. An approximate estimation in the analyzed population suggest a slow LD decay, comparable with those observed with the same markers set in a broader accessions panel [32]. The average extent of LD decay and localized chromosomal LD patterns in peach have also been estimated by genomic re-sequencing data, suggesting a faster decay, although only a small number of occidental breeding-derived accessions were included [28]. For such reason a conservative window size of about 200 Kbp was chosen to search for CGs around most significant regions. Inspecting ‘Kamarat’ and ‘Yumyeong’ re-sequencing data for the regions at about 9 Mb on chromosome 2, we identified two putative high-impact variants on the candidate *RTM2-like* gene (Prupe.2G065600). Association of the 5′-UTR variant with a reduced disease severity was confirmed in a panel of 73 accessions. As deduced by conceptual translation, PpRTM2-like (encoded by Prupe.2G065600) shares the same functional domains as the Arabidopsis RTM2 protein (AtRTM2), such as the conserved α-crystalline-like domain, a C-terminus transmembrane domain and the coiled-coils regions [48] (Fig. 7). AtRTM2 is expressed in phloem and sieve elements and has been associated to the specific restriction of the long-distance movement of TEV and other potyviruses, including PPV [36, 49], although the mechanism of action is still unknown. The expression pattern of the Prupe.2G065600 transcript was not assessed in this study, and, thus, the effect of the CT/GA-repeat deletion within the 5′-UTR remains to be elucidated. As demonstrated in several species, CT/GA motifs proximal to the ATG start codon play an important role in the regulation of gene expression ([50] and references therein). The 5′-UTR and exon II variants were also evaluated in progenies from ‘Orion’ (a susceptible cultivars) x ‘SDs’ crosses (PPV-resistant). However, at least in this genetic background, such mutations did not appear strongly associated with an increasing PPV tolerance or resistance, suggesting that genetic loci conferring quantitative tolerance in peach could be different from those conferring resistance in *P. davidiana*. As demonstrated in different studies with *P. davidiana* hybrids, the peach parent may affect the level of resistance in the progenies, and therefore, the identification of peach determinants could be also important for the introgression of a high level of resistance from related species. The variant found within *ENODL-like* gene (Prupe.3G291466) cannot be tested, since it is not present in *P. davidiana* or ‘Orion’ peach background.

In our study, the mapping resolution is mainly limited by the panel size, which does not allow the unequivocal identification of candidate gene(s) in the detected intervals. Despite this limitation, the loss-of-function mutation in the *ENODL-like* gene (Prupe.3G291466) or the several aminoacidic substitutions in *MYB33/65*-like (Prupe.2G050100) represents interesting candidates for future studies. For example, the miR159-regulated MYB33/65 plays a role in disease symptom induction by Cucumber Mosaic Virus in Arabidopsis [51]. The functions of *Nodulin-like* genes in non-nodulating species is still largely unknown, although recent studies highlight their importance in many aspects of plant development and plant-microbe interaction [52]. ENODL-like family members are supposed to be carbohydrate transporters, although some of them, such as AtENODL1,13–15, were differentially phosphorylated by the treatment with elicitors of plant immunity [53].

In perspective, an improvement of mapping resolution could be achieved by increasing markers density, for example through whole-genome re-sequencing. However, this approach could be advantageous only after the increase of sample size, identifying other tolerant/resistant genotypes and/or transferring the trait in different genetic backgrounds, primary in breeding-derived accessions.

## Conclusions

The present study is the first effort to identify genetic factors involved in Sharka disease in peach through a GWA approach. The understanding of the genetic basis of peach response to PPV infection is crucial to exploit favourable alleles already present in cultivated peach germplasm, representing a short-term solution for endemic areas and a more feasible approach compared to the introgression from related species. We provide evidence of the presence of quantitative resistant loci in a collection of peach accessions. Although with some limitations due to the small panel size and low number of tolerant individuals, we identified three major loci and three highly informative SNP markers, accounting for most of the phenotypic variability in PPV-M susceptibility that could be useful for marker assisted breeding or selection. Clearly, results should be confirmed by further studies. Biparental populations derived from this set of germplasm may represent a first step of validation and for this purpose, progenies derived from ‘Kamarat’ and ‘Ghiaccio1’ are presently being developed and will be directly evaluated by on-fields trials in endemic areas. Alternatively, the combination of association studies with larger populations and bi-parental linkage mapping could assist the confirmation of the identified loci and also the localization of additional loci or rare variants affecting host susceptibility.

## Methods

### Plant material and genotyping

The panel of accessions used in this study (Additional file 2) derived from some Italian peach germplasm collections**.** The IPSC peach 9 K SNP array [54] was used to genotype the analysed population of 85 individuals, using the SNPs selection criteria described in a previous study [32]. Genotyping data were filtered for marker missing rate < 10% and minor allele frequency (MAF) > 5%, finally retaining a total of 6009 SNPs for GWA analysis. The Peach Genome assembly V2.0 [55] was used as a reference for SNP marker positions.

### Phenotyping procedure

The degree of PPV susceptibility was evaluated through artificial inoculation, using the protocols described by Amenduni et al. [56]. Vegetative buds of each accession were grafted onto four to seven ‘GF305’ (peach seedlings) and inoculated by double chip-budding with PPV-M 0019 UBA, a highly virulent strain originated from Greece [57]. One or two plants for each accession were retained as healthy controls. The trial took place in insect-proof screenhouses located at the Centro di Ricerca e Sperimentazione in Agricoltura “Basile Caramia” (Locorotondo, Italy) or at the CRPV-Astra Martorano 5 (Cesena, Italy). Responses of the grafted scions to PPV infection were evaluated through visual inspection on a monthly basis, from early shoot growth until June, and for a minimum of two years. A scale-based scoring method taking into account symptoms intensity and distribution was adopted (Additional file 19: Figure S12), as follows: 0, no symptoms; 1, very light diffuse spots and symptoms in one or two leaves; 2, diffuse spots bordering leaf veins and symptoms in more than two leaves; 3, diffuse spots and deformed leaves, symptoms in most leaves. Symptomatic class was assigned each year based on the maximum degree of susceptibility showed along the time-points. The presence or absence of the virus was verified by ELISA assay using the universal monoclonal antibody 5B. Plants without symptoms on the shoots growing from the chip-buds or rootstocks and with a negative ELISA reaction for both the inoculum and rootstock were re-inoculated by chip-budding each year. Accessions negative to ELISA test were further assayed by RT-PCR as described by Wetzel et al. [58]. Among the 85 accessions used for GWA analysis, 46 were evaluated for PPV-M susceptibility (Additional file 1). The phenotypes of the remaining accessions were derived from studies adopting the same viral strain and evaluation protocol [59–61], except for a few cases of highly susceptible accessions derived from other publications [7, 8, 62]. Seedlings from three pseudo BC1 populations derived from ‘Orion’ (peach) x SD45, 75 and 81 (thress ‘Summergrand’ peach x *P. davidiana* ‘P1908’ selections) and composed of 39, 18 and 13 individuals, respectively, were evaluated using the same protocol described above. Each seedling was assigned to a symptomatic class after four years of observations (Additional file 17).

### Population genetic analysis

Population substructure was inferred in ADMIXTURE v1.22, a model-based clustering algorithm [63]. From SNP data, the software identifies K a priori genetic clusters provided by the user and for each individual estimates the probability of membership to each cluster. A preliminary analysis was performed by inputting successive values of K from 2 to 6. The value of K that maximized the predictive accuracy was chosen based on a 10-fold cross-validation procedure with 10 different fixed initial seeds. Principal Component Analysis (PCA) was also performed using the full set of filtered SNPs through the R function *prcomp*. The optimal number of PCs to be included for the considered phenotype were determined by using Bayesian information criterion (BIC). Phylogenetic tree was build from a pairwise genetic distance matrix between individuals, calculated as 1-IBS similarity, and clustered with UPGMA methods in TASSEL [64]. Bootstrap replicate and tree reconstruction was performed in MEGA6 software [65]. Linkage disequilibrium decay over distance was estimated by GAPIT, calculating r^2^ correlation for all pairwise SNPs comparisons on a sliding window with 100 adjacent markers. Intra-chromosomal LD patterns were measured and visualized using HAPLOVIEW v4.2 [66].

### Genome-wide association analysis

For association analysis, naïve Generalized Linear Model (GLM) and structured GLM using alternatively PCAs or Q matrix (calculated in ADMIXTURE) as covariates were performed in TASSEL; Mixed Linear Model (MLM), compressed MLM (CMLM) and Settlement of MLM Under Progressively Exclusion Relationship (SUPER) were conducted in GAPIT R package [67], involving EMMAX and P3D interfaces. Random effects were included in the mixed models as kinship matrix, either computed using Identical-By-State (IBS) and Balding-Nichols (BN) algorithms implemented in EMMAX package [68] or using the Van Raden algorithm (K), as implemented in GAPIT package. For fixed effects, either the first two PCs or Q-matrix (for K = 3) were used as covariates for association analysis. The Fixed and random model Circulating Probability Unification (FarmCPU) method was also tested [69]. FarmCPU separately estimates a fixed effects model using all tested markers and associated loci (pseudo-QTNs) and a random effects model using a kinship matrix defined by the pseudo-QTNs. Both effects models are used iteratively until no new pseudo-QTNs are added. The performance of all tested GWA algorithms was evaluated by comparing the observed vs expected *p*-values under null hypothesis through quantile-quantile (QQ) plot inspection and considering statistical power against False-Discovery Rate (FDR). A conservative threshold for assessing SNP significance was calculated based on Bonferroni correction for a type I error rate of 0.05. A two-stage approach was also tested, selecting SNP passing the FDR cut-off of 0.1 [70] and then fitting a logistic regression in PLINK [71]. To reduce small-sample bias in the maximum likelihood estimate in logit model, a penalized LASSO approach was applied in PUMA software, using AIC criterion for the choice of optimal lambda value [72]. Genetic model for putative locus-locus interactions were modelled by using the non-parametric Multifactor Dimensionality Reduction (MDR) 2.0 software [73]. The fitness of models was evaluated by assessing the cross-validation consistency (10-fold division of data) and testing accuracy (*p* ≤ 0.05).

### Variant identification from NGS data of tolerant/susceptible accessions

Whole-genome sequence (WGS) libraries of the accessions ‘Quetta’, ‘GF305’, ‘Yumyeong’, ‘Mayfire’, ‘Jing Yu’, ‘Venus’ and *P. davidiana* ‘P1908’ were retrieved from NCBI SRA archives (Additional file 20: Table S4). The library of ‘Kamarat’ was prepared by the Genomics Platform of Parco Tecnologico Padano (Lodi, Italy) with the Illumina TruseqDNA Nano sample prep kit (Illumina, San Diego) following manufacturer’s protocol and evaluated with the Agilent Tape Station 2200. The library was quantified with an ABI9700 qPCR instrument using the KAPA Library Quantification Kit in triplicates, according to the manufacturer’s protocol (Kapa Biosystems, Woburn, MA, USA). The Illumina Truseq PE cluster kit v3 was used to generate clusters on the grafted Illumina Flowcell and the hybridized molecules were sequenced on the Hiseq2000 with a 100 cycles of paired-end sequencing module using the Truseq SBS kit v3. FASTQ files were obtained with the Illumina’s CASAVA Pipeline. Reads were both sequence trimmed to remove the barcode and random hexamer and quality trimmed to remove low-quality bases. For variants detection, after adapter removal and quality filtering with Trimmomatic v0.32, reads were mapped onto peach reference genome V2.0 using BWA-MEM algorithm, implemented in BWA v.0.6.1 tool [74] using default parameters. An average coverage of 30.10× was estimated with Samtools mpileup tool. For SNP and short INDEL identification, after duplicate removal and reads indexing with PICARD, a joint-calling approach was performed using HC algorithm in GATK, following Best Practice guidelines. For identification of large indels, reads were realigned using RealignerTargetCreator and IndelRealigner tools, then filtered and merged, generating a single multi-sample file. Mapped reads were visualized in Tablet [75]. Variants were then annotated by using SNPEffect v2.0 [76] and peach reference genes annotations. For the prediction of candidate genes, the following priorities were considered: i) identification of variants from WGS; ii) genes with function-known orthologs in model plants species and related to plant-pathogen interaction; iii) genes pin-pointed by the peak SNPs.

### Marker validation and candidate variants analyses

Total genomic DNA was extracted from leaves of ‘Orion’ x ‘SD’ seedlings (derived from cross *P. davidiana* ‘P1908’ x ‘Summergrand’ peach) using a modified CTAB protocol and quantified using Qubit (ThermoFisher). Genomic DNA of the peach accessions listed in Table S4 was extracted using DNeasy Plant kit (Qiagen) following manufacturer’s instructions. The exon II and 5’ UTR variants in Prupe.2G065600, were genotyped through agarose gel-electrophoresis on ethidium-bromide 2% agarose gel. Amplicons were amplified in 10 ul Go-Taq reactions following the same conditions: 95 °C for 2 min, 35 cycles of 95 °C for 30 s, 58 °C for 30 s, and 72 °C for 1.5 min, with a final extension at 72^o^ C for 10 min. The three markers SNP_366639, SNP_214703 and SNP_185608 were scored through an HRMA-based approach. HRM analyses were carried out in an Eco Real-Time PCR System (Illumina, San Diego, USA) using 1X EVAGREEN Precision Melt Supermix (Bio-Rad, Hercules, USA). The reactions were carried out with the following programs: 2 min at 94 °C, 35 cycles of 30 s at 94 °C, 30 s annealing at 58 °C and 30 s at 72 °C, followed by a melting step over a 70–95 °C gradient with 0.1 °C/s ramp rate. Data were analyzed using EcoStudy software (Illumina, San Diego, USA). All primers are listed in Additional file 21: Table S5. Statistical significance of marker-trait associations were inferred using a non-parametric Kruskal-Wallis test.

## Additional files

Additional file 1: Phenotypic response to PPV-M infection evaluated in a panel of 73 peach accession using a 0 (asymptomatic) to 3 (severe) scale-based scoring method for symptoms intensity (XLSX 26 kb)

Additional file 2: List and classification of phenotypic response to PPV-M infection in the evaluated panel of accessions (XLSX 51 kb)

Additional file 3: Figure S1. Algorithms used for calculating the kinship matrix: A) Identical-By-State (IBS); B) Balding-Nichols (BN) and C) Van Raden (VR) (TIFF 288 kb)

Additional file 4: Figure S2. Scatter plot of the correlation between population structure and binary coded phenotypic values (tolerant vs susceptible) (TIFF 83 kb)

Additional file 5: Figure S3 Pattern of Linkage disequilibrium decay estimated from SNP array data (TIFF 107 kb)

Additional file 6: Figure S4. Manhattan and quantile-quantile plots of the -log10 *p*-values estimated for fruit flesh colour (top right panel) and fruit pubescence (bottom right panel) traits using FarmCPU algorithm. Red horizontal line indicates the Bonferroni-adjusted threshold based on the effective number of independent tests (−log10 2e-06) (TIFF 339 kb)

Additional file 7: Figure S5. Manhattan and quantile-quantile plots of the -log10 p-values estimated for binary (tolerant vs susceptible) coded phenotypic response to PPV infection in the panel of 85 accessions using Generalized Linear Model algorithm adjusted for population structure calculated through A) Q-matrix (for K = 3) and B) the first two principal component (PC1 and PC2). Red circle indicates significant SNP passing the Bonferroni-adjusted threshold (red horizontal line) based on the effective number of independent tests (−log10 2e-06) (TIFF 151 kb)

Additional file 8: Figure S6. Manhattan and quantile-quantile plots of the -log10 p-values estimated for binary (tolerant vs susceptible) coded phenotypic response to PPV infection in the panel of 85 accessions using A) Compressed Mixed Linear Model adjusted for kinship; B) Compressed Mixed Linear Model adjusted for kinship and population structure (Q-matrix for K = 3); C) SUPER model Red circle indicates significant SNPs passing the Bonferroni-adjusted threshold (red horizontal line) based on the effective number of independent tests (−log10 2e-06) (TIFF 223 kb)

Additional file 9: Full list of variants annotations and effects (as calculated with SNPEff tool) from the WGS libraries assembly of eight accession in the selected regions of chromosome 2 and 3. High-impact variants predicted by SNPEff are highlighted in red (XLSX 1522 kb)

Additional file 10: Figure S7. Annotated features of ‘Kamarat’ genome. Red, green and blue smoothed lines indicates gene density, nucleotide diversity (*pi*) and SNP density for each chromosome, respectively (TIFF 1757 kb)

Additional file 11: Figure S8. Linkage disequilibrium pattern around SNP_IGA_366639 on chromosome 3 (TIFF 506 kb)

Additional file 12: Table S1. List of candidate genes identified on the chromosome 3 region associated to the SNP_IGA_366639 (from 26.2 to 26.5 Mb) (DOCX 10 kb)

Additional file 13: Figure S9. Linkage disequilibrium pattern around SNP_IGA_214703 and SNP_IGA_218596 on chromosome 2 (TIFF 374 kb)

Additional file 14: Table S2. List of candidate genes identified on the chromosome 2 region associated to SNP_IGA_214703 (from 8.5 to 9.1 Mb) (DOCX 9 kb)

Additional file 15: Figure S10. Linkage disequilibrium pattern around SNP_IGA_185608 and SNP_IGA_185721 on chromosome 2 (TIFF 509 kb)

Additional file 16: Table S3. Candidate genes identified on the chromosome 2 associated to the SNP_IGA_185608 (from 5.6 to 6.0 Mb) (DOCX 9 kb)

Additional file 17: Phenotypic response to PPV-M infection in three pseudo BC1 progenies ‘Orion’ x ‘SD’ (‘Summergrand’ x *P. davidiana* ‘P1908’), evaluated using a 0 (asymptomatic) to 3 (severe) scale-based scoring method for symptoms intensity (XLSX 7 kb)

Additional file 18: Figure S11. Box-plots of single marker analysis for the 5′-UTR and the exon II variants inferred by the non-parametric Kruskal-Wallis K-test in 70 individuals from three pseudo BC1 progenies ‘Orion’ (peach) x SD (Summergrand x *P. davidiana* ‘P1908’) (TIFF 97 kb)

Additional file 19: Figure S12. Scale-based scoring method for evaluating plant response to PPV-M infection: class 0, no symptoms, ELISA and/or RT-PCR positive; class 1, very light diffuse spots, symptoms in one or two leaves; class 2, diffuse spots bordering leaf veins and symptoms in more than two leaves; class 3, diffuse spots and deformed leaves, symptoms in most leaves (TIFF 1658 kb)

Additional file 20: Table S4. SRA accession number (DOCX 8 kb)

Additional file 21: Table S5. List of primers used in this study. (DOCX 8 kb)

## Abbreviations

CMLM: Compressed mixed linear model
GLM: generalized linear model
GWA: genome-wide association
LD: Linkage disequilibrium
MAF: Minor allele frequency
MLM: Mixed linear model
PCA: Principal component analysis
PPV: plum pox virus
SNP: Single nucleotide polymorphism
WGS: whole-genome sequence
