# Supplementary figures and images for: Genetic dissection of Sharka disease tolerance in peach (P. persica L. Batsch)
Source: BMC Plant Biol. 2017 Nov 3;17:192. doi: 10.1186/s12870-017-1117-0 (PMC5670703; doi:10.1186/s12870-017-1117-0)

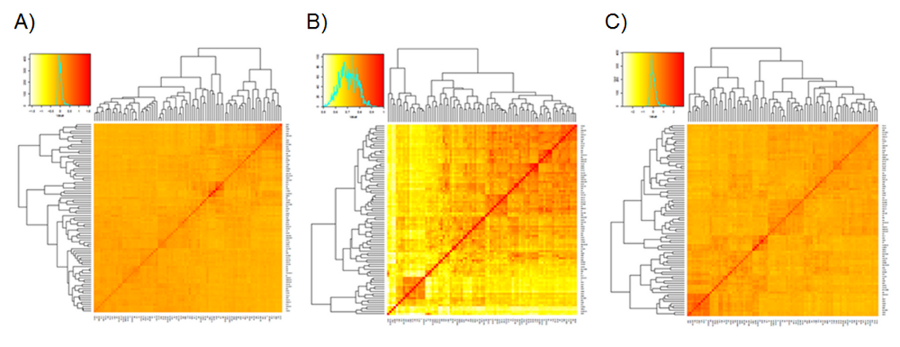

Supplement: Supplementary file 3 — Algorithms used for calculating the kinship matrix: A) Identical-By-State (IBS); B) Balding-Nichols (BN) and C) Van Raden (VR) (TIFF 288 kb) [file 12870_2017_1117_MOESM3_ESM.tif]

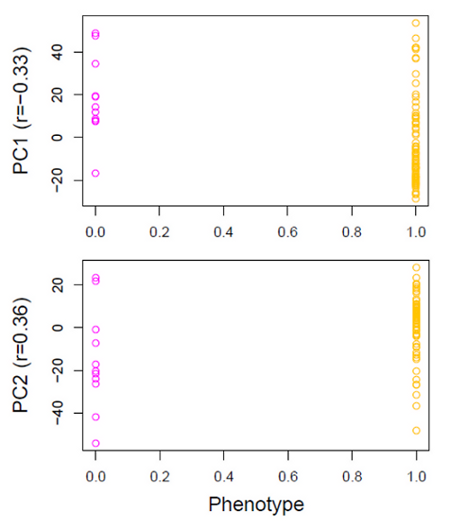

Supplement: Supplementary file 4 — Scatter plot of the correlation between population structure and binary coded phenotypic values (tolerant vs susceptible) (TIFF 83 kb) [file 12870_2017_1117_MOESM4_ESM.tiff]

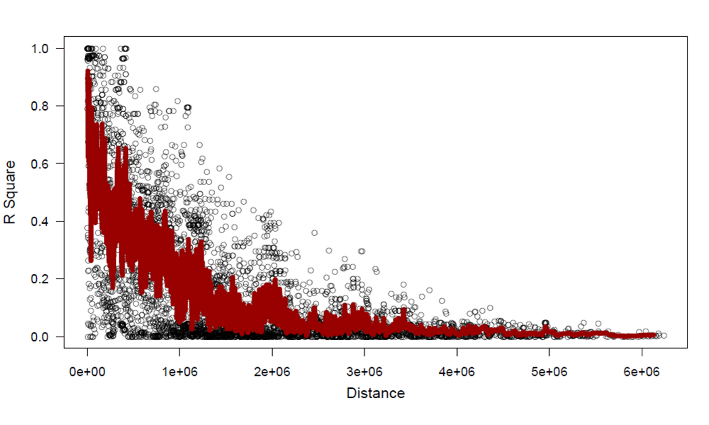

Supplement: Supplementary file 5 — Pattern of Linkage disequilibrium decay estimated from SNP array data (TIFF 107 kb) [file 12870_2017_1117_MOESM5_ESM.tiff]

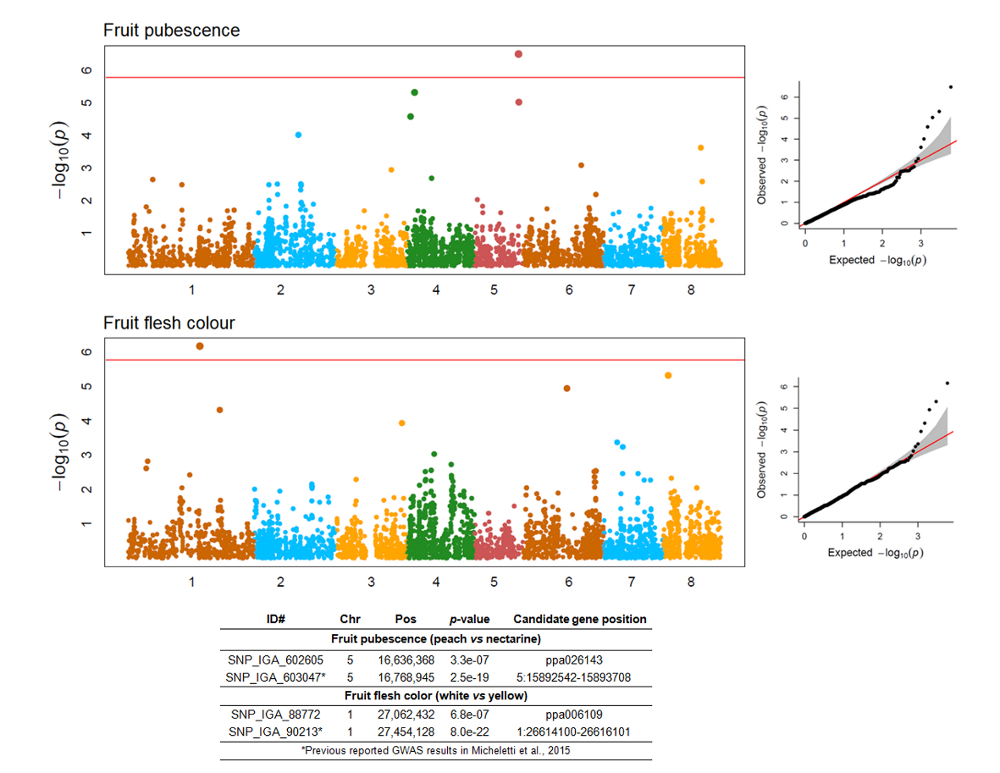

Supplement: Supplementary file 6 — Manhattan and quantile-quantile plots of the -log10 p-values estimated for fruit flesh colour (top right panel) and fruit pubescence (bottom right panel) traits using FarmCPU algorithm. Red horizontal line indicates the Bonferroni-adjusted threshold based on the effective number of independent tests (−log10 2e-06) (TIFF 339 kb) [file 12870_2017_1117_MOESM6_ESM.tiff]

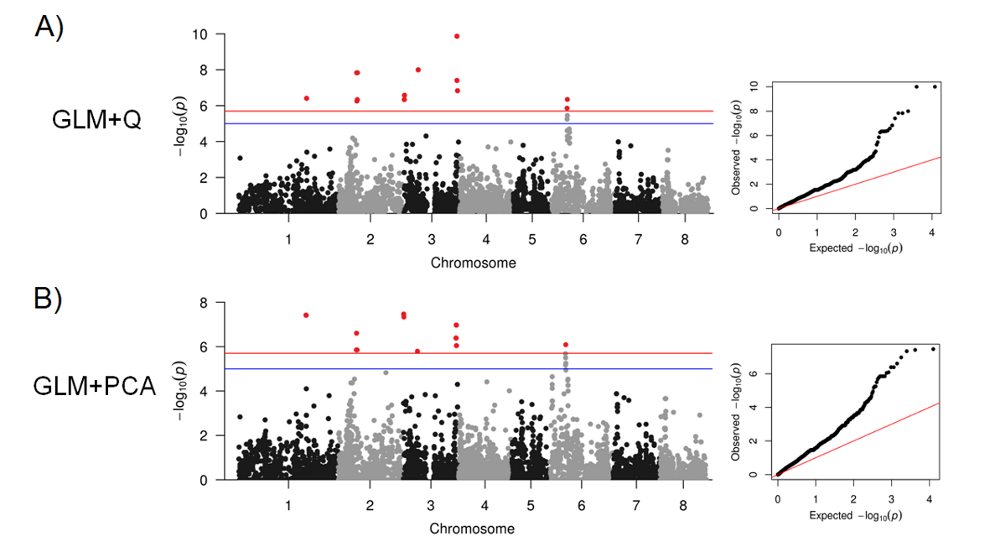

Supplement: Supplementary file 7 — Manhattan and quantile-quantile plots of the -log10 p-values estimated for binary (tolerant vs susceptible) coded phenotypic response to PPV infection in the panel of 85 accessions using Generalized Linear Model algorithm adjusted for population structure calculated through A) Q-matrix (for K = 3) and B) the first two principal component (PC1 and PC2). Red circle indicates significant SNP passing the Bonferroni-adjusted threshold (red horizontal line) based on the effective number of independent tests (−log10 2e-06) (TIFF 151 kb) [file 12870_2017_1117_MOESM7_ESM.tiff]

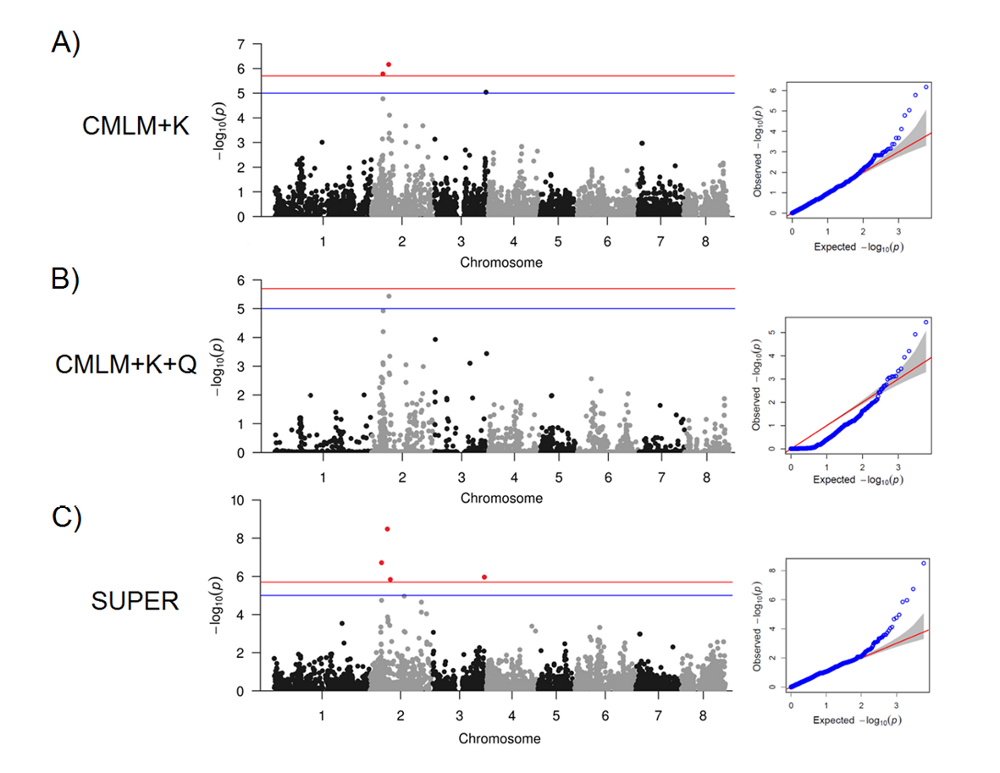

Supplement: Supplementary file 8 — Manhattan and quantile-quantile plots of the -log10 p-values estimated for binary (tolerant vs susceptible) coded phenotypic response to PPV infection in the panel of 85 accessions using A) Compressed Mixed Linear Model adjusted for kinship; B) Compressed Mixed Linear Model adjusted for kinship and population structure (Q-matrix for K = 3); C) SUPER model Red circle indicates significant SNPs passing the Bonferroni-adjusted threshold (red horizontal line) based on the effective number of independent tests (−log10 2e-06) (TIFF 223 kb) [file 12870_2017_1117_MOESM8_ESM.tiff]

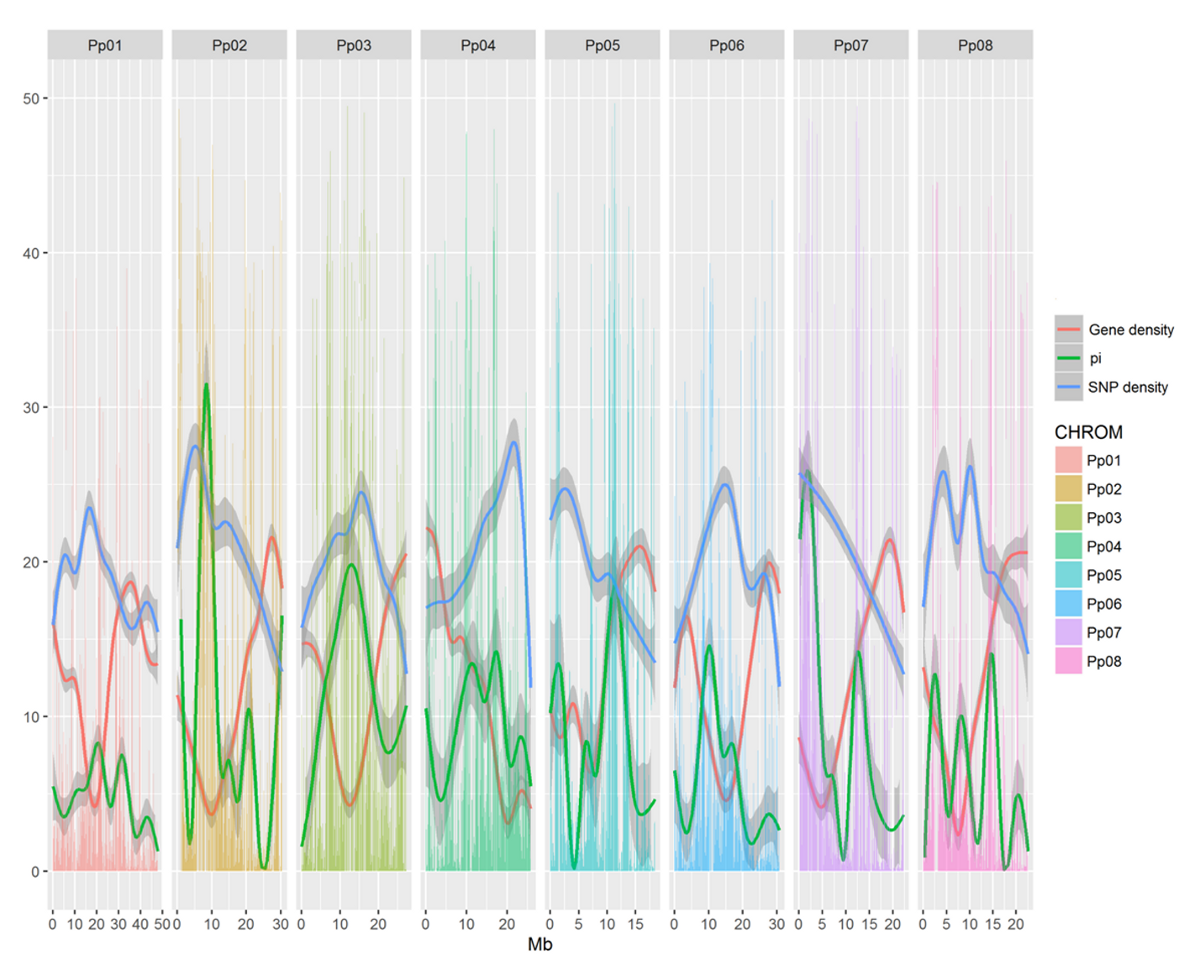

Supplement: Supplementary file 10 — Annotated features of ‘Kamarat’ genome. Red, green and blue smoothed lines indicates gene density, nucleotide diversity (pi) and SNP density for each chromosome, respectively (TIFF 1757 kb) [file 12870_2017_1117_MOESM10_ESM.tiff]

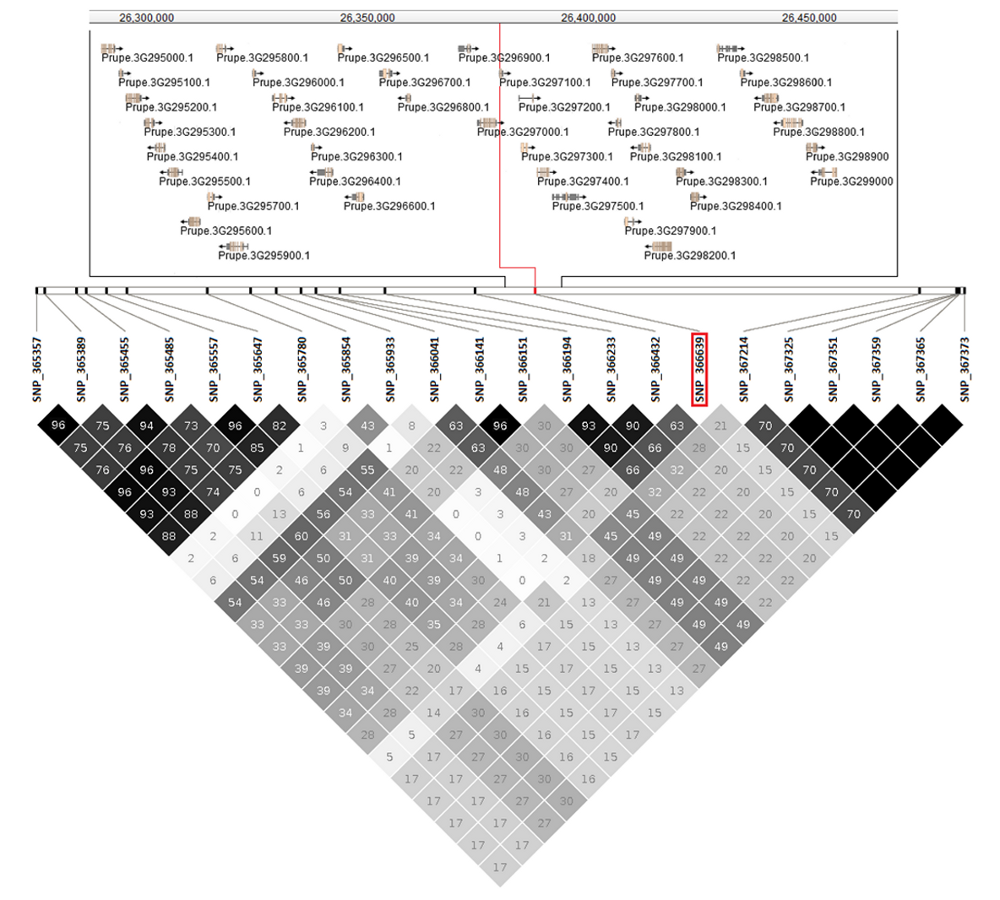

Supplement: Supplementary file 11 — Linkage disequilibrium pattern around SNP_IGA_366639 on chromosome 3 (TIFF 506 kb) [file 12870_2017_1117_MOESM11_ESM.tiff]

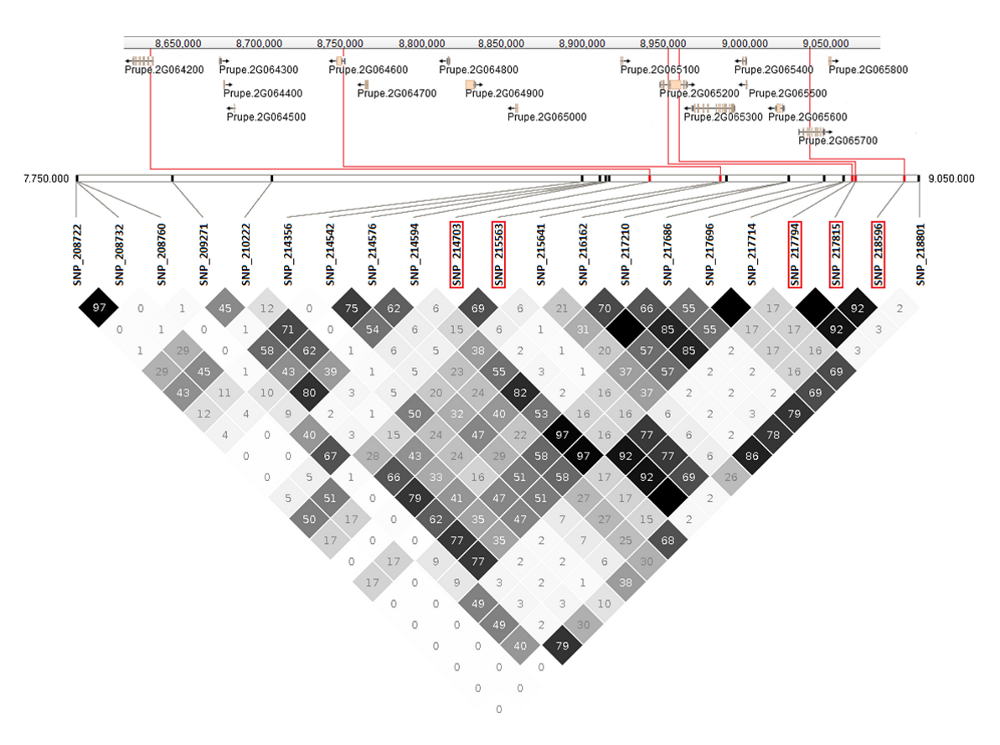

Supplement: Supplementary file 13 — Linkage disequilibrium pattern around SNP_IGA_214703 and SNP_IGA_218596 on chromosome 2 (TIFF 374 kb) [file 12870_2017_1117_MOESM13_ESM.tiff]

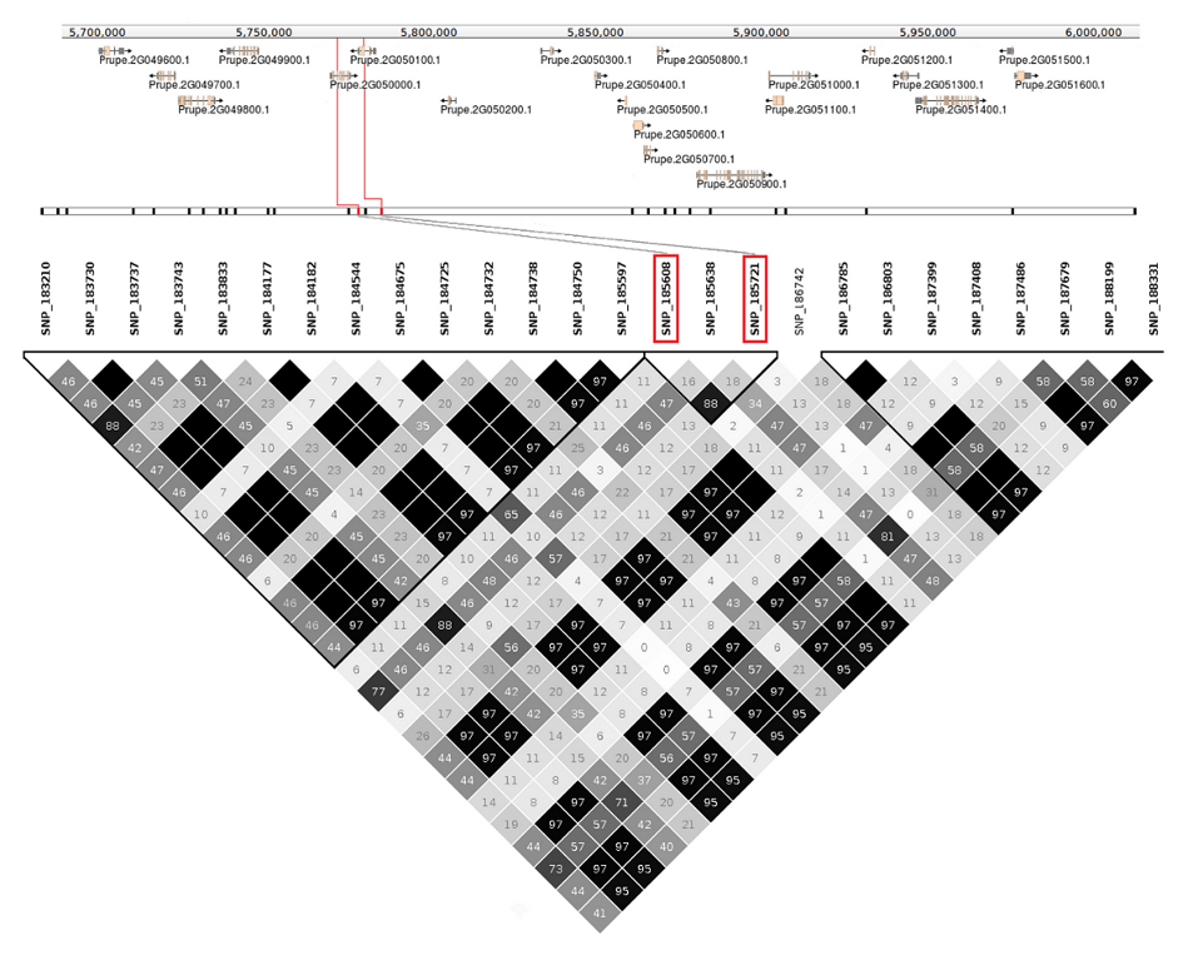

Supplement: Supplementary file 15 — Linkage disequilibrium pattern around SNP_IGA_185608 and SNP_IGA_185721 on chromosome 2 (TIFF 509 kb) [file 12870_2017_1117_MOESM15_ESM.tiff]

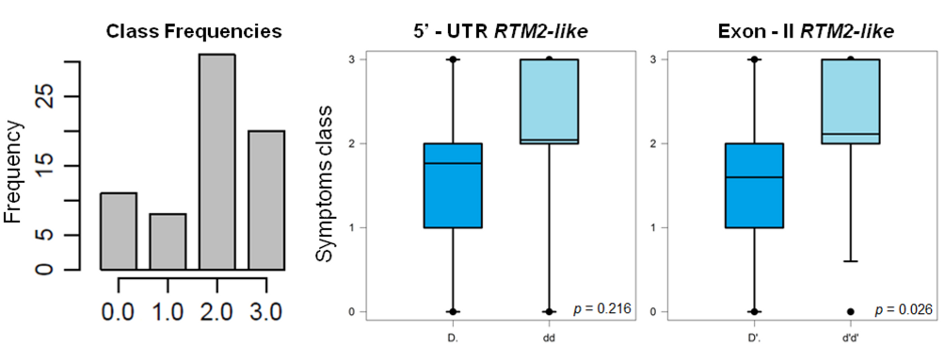

Supplement: Supplementary file 18 — Box-plots of single marker analysis for the 5′-UTR and the exon II variants inferred by the non-parametric Kruskal-Wallis K-test in 70 individuals from three pseudo BC1 progenies ‘Orion’ (peach) x SD (Summergrand x P. davidiana ‘P1908’) (TIFF 97 kb) [file 12870_2017_1117_MOESM18_ESM.tiff]

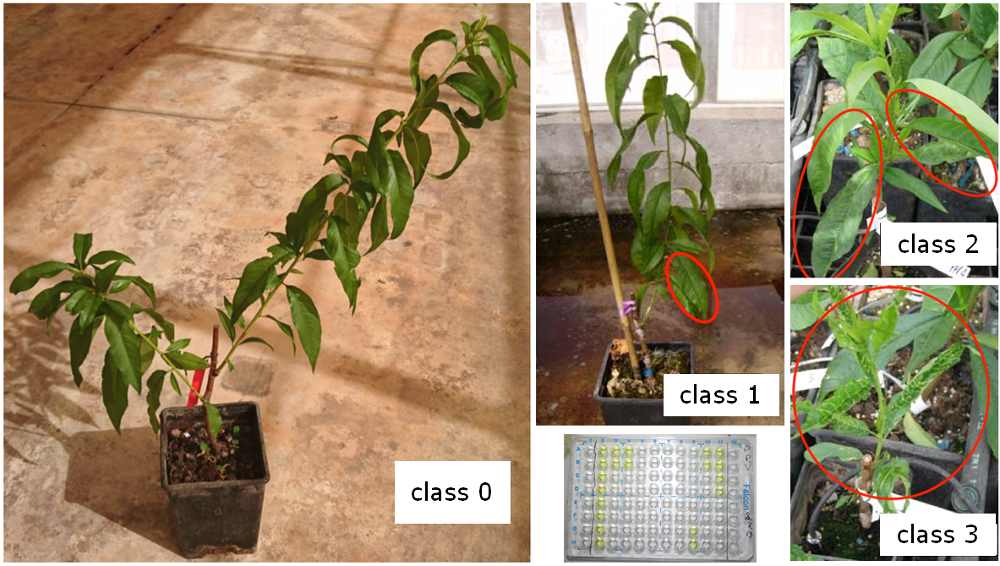

Supplement: Supplementary file 19 — Scale-based scoring method for evaluating plant response to PPV-M infection: class 0, no symptoms, ELISA and/or RT-PCR positive; class 1, very light diffuse spots, symptoms in one or two leaves; class 2, diffuse spots bordering leaf veins and symptoms in more than two leaves; class 3, diffuse spots and deformed leaves, symptoms in most leaves (TIFF 1658 kb) [file 12870_2017_1117_MOESM19_ESM.tiff]
