# Supplementary material for: Genetic dissection of Sharka disease tolerance in peach (P. persica L. Batsch)
Source: BMC Plant Biol. 2017 Nov 3;17:192. doi: 10.1186/s12870-017-1117-0 (PMC5670703; doi:10.1186/s12870-017-1117-0)
Supplement: Supplementary file 12 — List of candidate genes identified on the chromosome 3 region associated to the SNP_IGA_366639 (from 26.2 to 26.5 Mb) (DOCX 10 kb) [file 12870_2017_1117_MOESM12_ESM.docx]

**Supplemental Table 1.** Candidate genes on chromosome 3 from about 26.2 Mb to 26.5 Mb

| **Gene model** | **Position** | **A. thaliana homology** |
| --- | --- | --- |
| Prupe.3G294000 | Pp03:26253102..26256998 | DUF707 |
| Prupe.3G294100 | Pp03:26257815..26261271 | HISTONE ACETYLTRANSFERASE\| SGF29 |
| Prupe.3G294200 | Pp03:26262414..26263895 | AtRAB8D |
| Prupe.3G294300 | Pp03:26267829..26270513 | ZINC FINGER FYVE DOMAIN CONTAINING PROTEIN |
| Prupe.3G294400 | Pp03:26271432..26273777 | SURFEIT LOCUS PROTEIN 2 |
| Prupe.3G294500 | Pp03:26272889..26276820 | URACIL PHOSPHORIBOSYLTRANSFERASE |
| Prupe.3G294600 | Pp03:26277015..26281070 | METHYLTRANSFERASE PMT9-RELATED |
| Prupe.3G294700 | Pp03:26281440..26285957 | RING ZINC FINGER PROTEIN |
| Prupe.3G294800 | Pp03:26288517..26291665 | E3 UBIQUITIN-PROTEIN LIGASE RGLG1-RELATED |
| Prupe.3G294900 | Pp03:26292477..26293478 | UNKNOWN FUNCTION |
| Prupe.3G295000 | Pp03:26294012..26297631 | PPR REPEAT FAMILY |
| Prupe.3G295100 | Pp03:26298170..26300608 | RNA RECOGNITION MOTIF\| RRM1 |
| Prupe.3G295200 | Pp03:26300671..26302970 | RAS-RELATED PROTEIN RABB1B |
| Prupe.3G295300 | Pp03:26303483..26306939 | AT05866P-RELATED proteasome |
| Prupe.3G295400 | Pp03:26308185..26310786 | CYTOCHROME P450 |
| Prupe.3G295500 | Pp03:26312538..26314185 | METHYLTRANSFERASE |
| Prupe.3G295600 | Pp03:26314548..26316770 | SPLICING FACTOR 3b, SUBUNIT 4\| AtRBP31 |
| Prupe.3G295700 | Pp03:26316870..26321700 | BROMODOMAIN EXTRA-TERMINAL (BET) |
| Prupe.3G295800 | Pp03:26322717..26323513 | DUF1713) |
| Prupe.3G295900 | Pp03:26327165..26330549 | ALPHA/BETA-HYDROLASES SUPERFAMILY PROTEIN |
| Prupe.3G296000 | Pp03:26331534..26334722 | GLYCEROL-3-PHOSPHATE DEHYDROGENASE |
| Prupe.3G296100 | Pp03:26335983..26336768 | UNKNOWN FUNCTION |
| Prupe.3G296200 | Pp03:26337406..26340864 | KUB3-PROV PROTEIN Ku70 |
| Prupe.3G296300 | Pp03:26341978..26343576 | UNKNOWN FUNCTION |
| Prupe.3G291450 | Pp03:26345295..26347881 | PECTATE LYASE 11-RELATED |
| Prupe.3G291451 | Pp03:26351442..26354405 | CCT MOTIF (CCT) |
| Prupe.3G291452 | Pp03:26357343..26358464 | UBIQUITIN |
| **Prupe.3G291466** | **Pp03:26378550..26379486** | **EARLY NODULIN-LIKE PROTEIN 21** |
| Prupe.3G291467 | Pp03:26390557..26396553 | E3 UBIQUITIN-PROTEIN LIGASE RNF115 |
| Prupe.3G291468 | Pp03:26403944..26404846 | EARLY NODULIN-LIKE PROTEIN 21 |
| Prupe.3G291469 | Pp03:26404939..26406104 | CLEAVAGE SITE TYPE III EFFECTOR AVIRULENCE FACTOR |
| Prupe.3G291470 | Pp03:26406922..26409078 | SUCCINATE DEHYDROGENASE IRON-SULFUR SUBUNIT |
| Prupe.3G291471 | Pp03:26409999..26412839 | ATP SYNTHASE MITOCHONDRIAL F1 COMPLEX |
| Prupe.3G291472 | Pp03:26413287..26417549 | BETA-GALACTOSIDASE 1 |
| Prupe.3G291473 | Pp03:26418649..26420879 | CYSTINOSIN |
| Prupe.3G291474 | Pp03:26421825..26423825 | CYSTINOSIN |
| Prupe.3G291475 | Pp03:26427909..26432388 | CYSTEIN TRANSPORTER |
| Prupe.3G291476 | Pp03:26433100..26434267 | CYSTEIN TRANSPORTER |
| Prupe.3G291477 | Pp03:26438027..26441831 | TPX2 (TARGETING PROTEIN FOR XKLP2) PROTEIN FAMILY |
| Prupe.3G291478 | Pp03:26442355..26447371 | PEPTIDYL SERINE ALPHA-GALACTOSYLTRANSFERASE |
| Prupe.3G291479 | Pp03:26448088..26450572 | ZINC FINGER PROTEIN-RELATED |
| Prupe.3G291480 | Pp03:26450861..26454916 | MYB TRANSCRIPTION FACTOR |
| Prupe.3G291481 | Pp03:26458666..26462899 | C3H4 TYPE ZINC FINGER PROTEIN-RELATED |
| Prupe.3G291482 | Pp03:26463362..26471931 | GPI INOSITOL-DEACYLASE |
| Prupe.3G291483 | Pp03:26472511..26475722 | CARBOXYLESTERASE 11-RELATED |
| Prupe.3G291484 | Pp03:26476968..26481090 | SERINE/THREONINE PROTEIN PHOSPHATASE 2A |
