# Supplementary material for: Genetic dissection of Sharka disease tolerance in peach (P. persica L. Batsch)
Source: BMC Plant Biol. 2017 Nov 3;17:192. doi: 10.1186/s12870-017-1117-0 (PMC5670703; doi:10.1186/s12870-017-1117-0)
Supplement: Supplementary file 14 — List of candidate genes identified on the chromosome 2 region associated to SNP_IGA_214703 (from 8.5 to 9.1 Mb) (DOCX 9 kb) [file 12870_2017_1117_MOESM14_ESM.docx]

**Supplemental Table 2.** Candidate genes on chromosome 2 from about 8.5 Mb to 9.1 Mb

| **Gene** | **Position** | **A. thaliana homology** |
| --- | --- | --- |
| Prupe.2G063700 | Pp02:8547981..8548699 | Ubl1\| SUMO Sentrin |
| Prupe.2G063800 | Pp02:8561919..8563066 | CDF2\| cycling DOF factor 2 |
| Prupe.2G063900 | Pp02:8575471..8581528 | Tyrosine kinase\| Leucine rich repeat (LRR8) |
| Prupe.2G064000 | Pp02:8584755..8586945 | unknown function |
| Prupe.2G064100 | Pp02:8588079..8590108 | PPR superfamily protein |
| Prupe.2G064200 | Pp02:8634291..8640748 | NOL\| Chlorophyll(ide) b reductase |
| Prupe.2G064300 | Pp02:8673702..8674563 | unknown function |
| Prupe.2G064400 | Pp02:8675023..8675438 | H3 LYSINE-9 SPECIFIC SUVH5-RELATED |
| Prupe.2G064500 | Pp02:8678318..8678524 | unknown function |
| Prupe.2G064600 | Pp02:8747500..8749952 | H3 LYSINE-9 SPECIFIC SUVH5-RELATED |
| Prupe.2G064700 | Pp02:8756168..8757229 | GAG-POL LTR Copia-type |
| Prupe.2G064800 | Pp02:8831477..8832260 | unknown function |
| Prupe.2G064900 | Pp02:8837323..8840389 | Protein kinase\| Leucine rich repeat (LRRNT2) |
| Prupe.2G065000 | Pp02:8852887..8853781 | GAG-POL related RETROTRANSPOSON |
| Prupe.2G065100 | Pp02:8935412..8936224 | unknown function |
| Prupe.2G065200 | Pp02:8947552..8956125 | PHOSPHOINOSITIDE-3-KINASE REGULATORY SUBUNIT 4 |
| Prupe.2G065300 | Pp02:8957344..8970685 | DEA(D/H)-box RNA helicase family protein |
| Prupe.2G065400 | Pp02:8993887..8995033 | unknown function |
| **Prupe.2G065600** | **Pp02:9010591..9013440** | **RTM2\| HSP20 FAMILY SMALL HEAT-SHOCK protein** |
| Prupe.2G065700 | Pp02:9024001..9032210 | Protodioscin-26-O-beta-D-glucosidase |
| Prupe.2G065800 | Pp02:9050564..9050902 | E3 ubiquitin ligase SCF complex SKP1/ASK1 family protein |
| Prupe.2G065900 | Pp02:9052888..9054539 | PSRP5\|plastid-specific 50S ribosomal protein 5 |
| Prupe.2G066000 | Pp02:9077117..9083604 | PPR superfamily protein |
| Prupe.2G066100 | Pp02:9109305..9110954 | DET2\| 3-oxo-5-alpha-steroid-4-dehydrogenase |
| Prupe.2G066200 | Pp02:9111548..9129074 | UBIQUITIN CARBOXYL-TERMINAL HYDROLASE 15 |
