# Supplementary material for: Genetic dissection of Sharka disease tolerance in peach (P. persica L. Batsch)
Source: BMC Plant Biol. 2017 Nov 3;17:192. doi: 10.1186/s12870-017-1117-0 (PMC5670703; doi:10.1186/s12870-017-1117-0)
Supplement: Supplementary file 16 — Candidate genes identified on the chromosome 2 associated to the SNP_IGA_185608 (from 5.6 to 6.0 Mb) (DOCX 9 kb) [file 12870_2017_1117_MOESM16_ESM.docx]

**Supplemental Table 3.** Candidate genes on chromosome 2 from about 5.6 Mb to 6.0 Mb

| **Gene** | **Position** | **A. thaliana homology** |
| --- | --- | --- |
| Prupe.2G049200.1 | Pp02:5659348..5661571 | Nascent polypeptide-associated complex (NAC) |
| Prupe.2G049400.1 | Pp02:5663117..5664066 | Mitochondrial transcription termination factor family protein |
| Prupe.2G049500.1 | Pp02:5664325..5666286 | SAC3/GANP/Nin1/mts3/eIF-3 p25 family |
| Prupe.2G049800.1 | Pp02:5723000..5734079 | SUA \| suppressor of abi3-5 |
| Prupe.2G049900.1 | Pp02:5737554..5747153 | DET1, FUS2, ATDET1 \| light-mediated development protein |
| **Prupe.2G050000.1** | **Pp02:5768759..5774832** | **Protein kinase superfamily protein** |
| **Prupe.2G050100.1** | **Pp02:5777118..5782601** | **AtMYB33 \| myb domain protein 33** |
| Prupe.2G050300.1 | Pp02:5832216..5836205 | Nucleotide-diphosphosugar transferases family protein |
| Prupe.2G050400.1 | Pp02:5848437..5849997 | AtCNGC1 \| cyclic nucleotide gated channel 1 |
| Prupe.2G050800.1 | Pp02:5867191..5868718 | NAD(P)-linked oxidoreductase superfamily protein |
| Prupe.2G050900.1 | Pp02:5879031..5899698 | ARM repeat superfamily protein |
| Prupe.2G051000.1 | Pp02:5900796..5913485 | Polynucleotidyl transferase, ribonuclease H-like family protein |
| Prupe.2G051100.1 | Pp02:5902195..5905326 | NB-ARC domain-containing disease resistance protein |
| Prupe.2G051200.1 | Pp02:5931183..5932759 | UBP12 \| ubiquitin-specific protease 12 |
| Prupe.2G051400.1 | Pp02:5945009..5963952 | ARM repeat superfamily protein |
| Prupe.2G051500.1 | Pp02:5972467..5974452 | Alpha-xylosidase |
| Prupe.2G051600.1 | Pp02:5974702..5979642 | Pentatricopeptide repeat (PPR) superfamily protein |
