# Supplementary material for: Genetic dissection of Sharka disease tolerance in peach (P. persica L. Batsch)
Source: BMC Plant Biol. 2017 Nov 3;17:192. doi: 10.1186/s12870-017-1117-0 (PMC5670703; doi:10.1186/s12870-017-1117-0)
Supplement: Supplementary file 20 — SRA accession number (DOCX 8 kb) [file 12870_2017_1117_MOESM20_ESM.docx]

**Supplemental Table 4**. SRA accession number of assembled Illumina Whole-Genome libraries.

| **accession** | **SRR** | **SAM** | **SRX** | **Systematic Name** | |
| --- | --- | --- | --- | --- | --- |
| GF305 | SRR502983 | SAMN01000700 | SRX150234 | *P. persica* (L.) Batsch. | |
| Jing Yu | SRR830547 | SAMN02048762 | SRX268357 | *P. persica* (L.) Batsch. | |
| Mayfire | SRR830548 | SAMN02048763 | SRX268358 | *P. persica* (L.) Batsch. | |
| Quetta | SRR502987 | SAMN01000704 | SRX150241 | *P. persica* (L.) Batsch. | |
|  | SRR502989 |  | SRX150243 | *P. persica* (L.) Batsch. | |
| Venus | SRR1867740 | SAMN03393052 | SRX906265 | *P. persica* (L.) Batsch. | |
|  | SRR1867794 |  |  |  |  |
|  | SRR1867795 |  |  |  |  |
| Yumyeong | SRR502994 | SAMN01000707 | SRX150253 | *P. persica* (L.) Batsch. | |
| *P. davidiana* Cl. P1908 | SRR502982 | SAMN01000695 | SRX150227 | *P. davidiana* (Carr.) Franch. | |
| Kamarat | Under submission | | | | *P. persica* (L.) Batsch |
