# Supplementary material for: Genetic dissection of Sharka disease tolerance in peach (P. persica L. Batsch)
Source: BMC Plant Biol. 2017 Nov 3;17:192. doi: 10.1186/s12870-017-1117-0 (PMC5670703; doi:10.1186/s12870-017-1117-0)
Supplement: Supplementary file 21 — List of primers used in this study. (DOCX 8 kb) [file 12870_2017_1117_MOESM21_ESM.docx]

**Supplemental Table 5**. Primer list for genotyping selected markers and candidate variants.

| **Primer name** | **Sequence** |
| --- | --- |
| **Locus SNP_ 366639** | |
| SNP366639_For | GTTTTGGCCATTTCTCAGTCTCTG |
| SNP366639_Rev | GCTGTCATTAGTAGGAGGCACAAC |
| **Locus SNP_ 214703** | |
| SNP214703_For | AGGCTCTGGACAGCAGATTTAAC |
| SNP214703_Rev | ATTGGCTGATGGAAGTTGTGACT |
| **Locus SNP_ 185608** | |
| SNP185608_For | CCCTGGAGCAACTTTCTCTAAATT |
| SNP185608_Rev | GTTGTTTGCATGCAGGTATTGTAA |
| **Prupe.2G065600 (RTM2-like)** | |
| **5' UTR variant** | |
| RTM2-like_UTR_For | TATAACGTGAGCTTCCTGCTCTTC |
| RTM2-like_UTR_Rev | TGTTTGCATGAGACCGTTATACCTC |
| **Exon II variant** | |
| RTM2-like_CDS_For | TCATTCTGGATTGCTGAAATTGCT |
| RTM2-like_CDS_Rev | AAAGCAAAGGGATAAGAGAAGTGC |
